# Natural variation in *SlGRF10* reveals a role in regulating tomato fruit weight

**DOI:** 10.1093/plphys/kiag465

**Published:** 2026-07-01

**Authors:** Julia von Steimker, Markéta Macho, Regina Wendenburg, Jeongah Lee, Itay Zemach, Yimin Xu, Anja Fröhlich, Arun Sampathkumar, Dani Zamir, Zhangjun Fei, James J Giovannoni, Alisdair R Fernie, Saleh Alseekh

**Affiliations:** Max-Planck-Institute of Molecular Plant Physiology, Am Mühlenberg 1, Potsdam-Golm 14476, Germany; Max-Planck-Institute of Molecular Plant Physiology, Am Mühlenberg 1, Potsdam-Golm 14476, Germany; Max-Planck-Institute of Molecular Plant Physiology, Am Mühlenberg 1, Potsdam-Golm 14476, Germany; Max-Planck-Institute of Molecular Plant Physiology, Am Mühlenberg 1, Potsdam-Golm 14476, Germany; Robert H. Smith Institute of Plant Sciences and Genetics, Faculty of Agriculture, Hebrew University of Jerusalem, Rehovot 7610001, Israel; Boyce Thompson Institute for Plant Research, Cornell University, Ithaca, NY 14853, United States; Max-Planck-Institute of Molecular Plant Physiology, Am Mühlenberg 1, Potsdam-Golm 14476, Germany; Max-Planck-Institute of Molecular Plant Physiology, Am Mühlenberg 1, Potsdam-Golm 14476, Germany; Robert H. Smith Institute of Plant Sciences and Genetics, Faculty of Agriculture, Hebrew University of Jerusalem, Rehovot 7610001, Israel; Boyce Thompson Institute for Plant Research, Cornell University, Ithaca, NY 14853, United States; Boyce Thompson Institute for Plant Research, Cornell University, Ithaca, NY 14853, United States; US Department of Agriculture/Agriculture Research Service, Robert W. Holley Centre for Agriculture and Health, Ithaca, NY 14853, United States; Max-Planck-Institute of Molecular Plant Physiology, Am Mühlenberg 1, Potsdam-Golm 14476, Germany; Center of Plant Systems Biology and Biotechnology, Plovdiv 4023, Bulgaria; Max-Planck-Institute of Molecular Plant Physiology, Am Mühlenberg 1, Potsdam-Golm 14476, Germany; Center of Plant Systems Biology and Biotechnology, Plovdiv 4023, Bulgaria

## Abstract

Fruit weight is a key determinant of yield in high-value vegetable crops such as tomato. Despite extensive research, the molecular mechanisms underlying this complex trait remain largely elusive, with only a few genes cloned to date based on quantitative trait loci (QTL). Here, we analyzed 2 populations and reanalyzed 3 previously published populations and identified 945 QTL associated with agro-morphological traits, including both previously reported and unidentified loci. We focused on *SlGRF10* (*GROWTH-REGULATING FACTOR 10*) underlying a fruit weight QTL, *fw1.2*. Loss of *SlGRF10* reduced fruit weight by decreasing cell size, without affecting cell number. Analysis of natural variation in *SlGRF10* in over 1,000 tomato accessions revealed that increased single-nucleotide polymorphism diversity in *SlGRF10* is associated with lower fruit weight. This suggests that putative impaired activity contributes to reduced fruit weight, while breeding-induced reduction of genetic variation may have promoted increased fruit weight. Transcriptome profiling of *SlGRF10* knockout lines 7 and 20 days postanthesis identified several differentially expressed genes involved in cell cycle progression. Our findings not only confirm the role of *SlGRF10* in regulating tomato fruit weight but also highlight a set of candidate genes associated with key morpho-physiological traits. With *fw11.3*, named as *CELL SIZE REGULATOR*, being the only QTL related to cell size determination in tomato fruits so far, *SlGRF10* offers a valuable target for precision breeding and enhances our understanding of fruit weight in tomato and related fruit-bearing species.

## Teaser

A growth-regulating gene shapes tomato fruit weight by controlling cell expansion, offering new opportunities to improve crop yield through precision breeding.

## Introduction

Tomato (*Solanum lycopersicum*) is one of the world's most important vegetable crops from an agronomic perspective and has also served as a model species for studying fleshy fruit physiology and development (Butelli et al. 2008; Martin et al. 2011; Tieman et al. 2017; Goldman et al. 2023). Domestication and diversification have resulted in substantial phenotypic variation between cultivated tomato varieties and their wild ancestors, affecting traits such as yield, fruit shape, fruit size, and other agronomic characteristics (Meyer and Purugganan 2013; Fuller et al. 2014; Goldman et al. 2023). Understanding the genetic basis of yield-related traits remains a central goal in tomato breeding.

To date, numerous quantitative trait locus (QTL) studies have identified many loci linked to fruit weight and yield, indicating a complex genetic architecture underlying these traits (Eshed and Zamir 1995; Grandillo et al. 1999; Causse et al. 2002; Illa-Berenguer et al. 2015; Barrantes et al. 2016; Pereira et al. 2021). Fruit weight is ultimately governed by the size and organization of the floral meristem, as well as by cell proliferation, cell division, and cell expansion (van der Knaap et al. 2014). Despite extensive efforts, only a few fruit weight-related genes have been cloned thus far. Among these, *fw2.2*, encoding *CELL NUMBER REGULATOR*, and *fw3.2*, encoding *SlKLUH*, regulate fruit size primarily through cell division (Frary et al. 2000; Chakrabarti et al. 2013), with the latter demonstrating conserved function across diverse plant species (Anastasiou et al. 2007; Adamski et al. 2009; Stransfeld et al. 2010; Jiang et al. 2021). By contrast, *fw11.3*, encoding *CELL SIZE REGULATOR*, influences cell expansion (Mu et al. 2017). Other key genes such as *locule number* (*lc*) (Muños et al. 2011) and *fasciated* (*fas*) (Cong et al. 2008; Xu et al. 2015) are linked to locule number but also contribute to variation in fruit weight. Pereira et al. (2021) identified 6 additional QTL associated with fruit weight, *fw1.1*, *fw2.3*, *fw3.3*, *fw7.1*, *fw8.1*, and *fw11.2*, based on F_2_ populations.

Building on this foundation, we recently screened a global collection of over 7,900 tomato accessions and identified associations with several complex traits, including fruit weight and total soluble solids (Brix) (Zemach et al. 2023). Among the findings, we uncovered a gene-trait association implicating *SlGRF10* (*fw1.2*), a growth-regulating factor (GRF), in the control of fruit weight. GRFs are plant-specific transcription factors that regulate growth, development, and shelf life (Bao et al. 2014; Debernardi et al. 2014; Liu et al. 2014; Omidbakhshfard et al. 2015). These proteins contain 2 conserved domains: the QLQ domain (glutamine-leucine-glutamine), which mediates interactions with GRF-interacting factors (GIFs), and the WRC domain (tryptophan-arginine-cysteine), responsible for DNA binding and nuclear localization (Van der Knaap et al. 2000; Kim et al. 2003; Zhang et al. 2008). Despite their importance, GRFs have been relatively understudied in tomato, though they are known to regulate leaf, flower, and fruit development (Cao et al. 2016; Khatun et al. 2017; Omidbakhshfard et al. 2018; Mauxion et al. 2021).

In the present study, we investigated the genetic architecture of *SlGRF10* through Clustered Regularly Interspaced Short Palindromic Repeats-associated protein 9 (CRISPR-Cas9) knockout experiments combined with transcriptomic analysis. Analysis of natural variation across multiple accessions further revealed a correlation between high nucleotide diversity in *SlGRF10* and reduced fruit weight. Functional characterization revealed that *SlGRF10* influences fruit weight by affecting cell expansion, but not total cell number. Together, our findings provide insights into the complex regulation of fruit weight and establish *SlGRF10* as a valuable genetic resource for improving yield in tomato and potentially other fruit crops.

## Results

### Natural variation of agro-morphological traits in tomato

**Figure 1:**
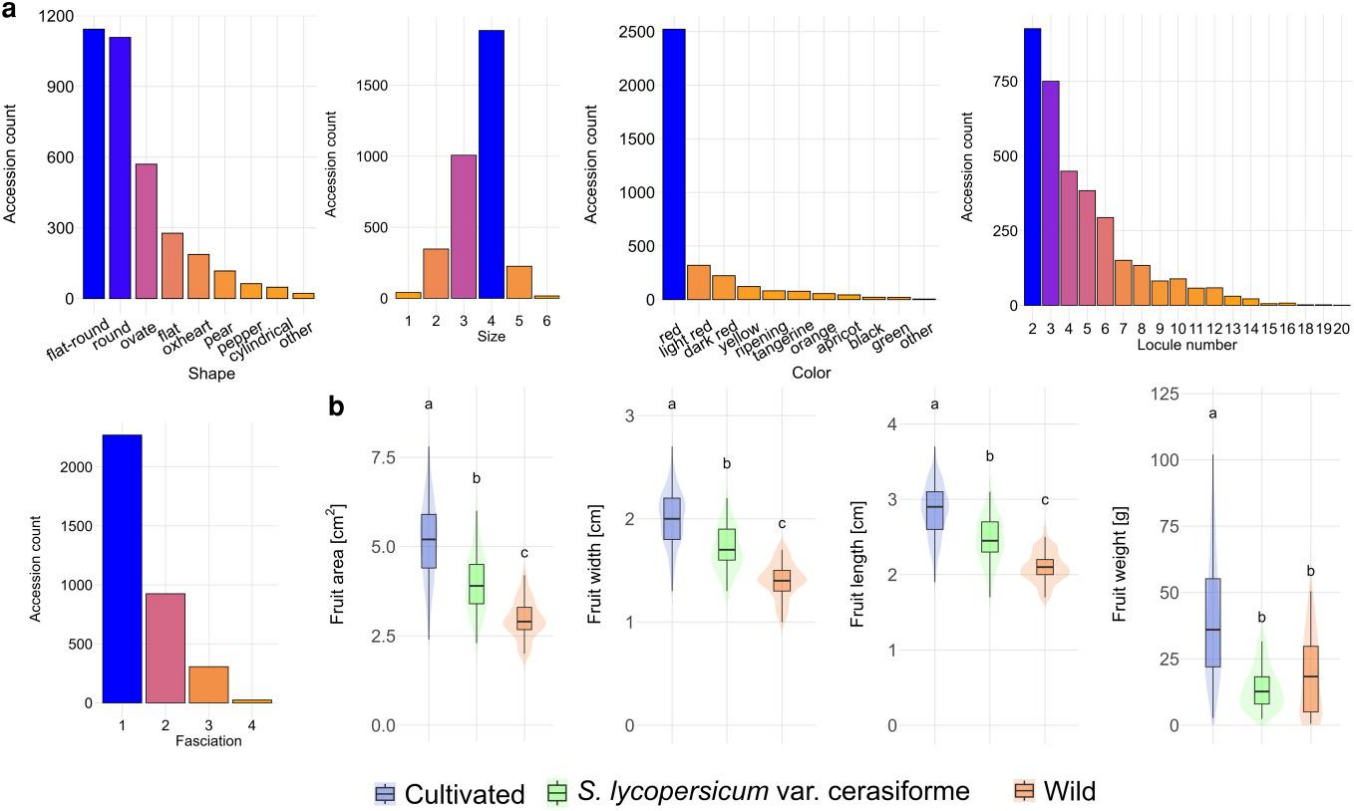
Morphological descriptors of the multiyear field trial. a) Fruit shape, size (1, very small; 2, small; 3, medium-small; 4, medium; 5, large; 6, very large), color, locule number, and fasciation (1, absent; 2, slight; 3, moderate; 4, strong) are shown as bar plots of the full 3,591 panel. b) Box plots of fruit area, length, width, and weight of cultivated *S. lycopersicum* (*n* = 436), *S. lycopersicum* var. *cerasiforme*, (*n* = 84), and wild species (*n* = 97). Letters indicate significance *P* < 0.001, Kruskal–Wallis test, post hoc Dunn's test. Updated based on Zemach et al. (2023). Boxplots show median, interquartile range (IQR), and 1.5× IQR whiskers.

Shape-related features such as fruit weight, size, color, fasciation, and locule number are key determinants of consumer preferences in vegetable crops like tomato (Goldman et al. 2023). In a recent study, we analyzed a global genetic resource collection comprising over 7,900 tomato accessions (Zemach et al. 2023). This collection displays remarkable diversity in fruit morphology, including shape, size, and color. Among a core diversity panel of 3,591 accessions, flat-round to ovate fruit shapes, medium-sized red fruits, single fasciation, and double to triple locule numbers are predominant (Fig. 1a).

To further characterize this variation, we examined fruit shape-related traits in a panel of 674 tomato accessions, integrating phenotypic measurements with single-nucleotide polymorphism (SNP) genotype data. From an initial population of 3,591 accessions, lines exhibiting poor germination, incomplete phenotypic records, or inconsistent measurements across replicates were excluded. The final set of 674 accessions was selected by Zemach et al. 2023 to maximize phenotypic and genetic diversity while ensuring robustness for downstream analyses. This analysis confirmed a significantly greater fruit area, length, width and weight in cultivated *S. lycopersicum* compared with *S. lycopersicum* var. *cerasiforme* and wild relatives (Lin et al. 2014; van der Knaap et al. 2014) (Figs. 1b and  S1).

### Genetic architecture of tomato fruit weight

**Figure 2:**
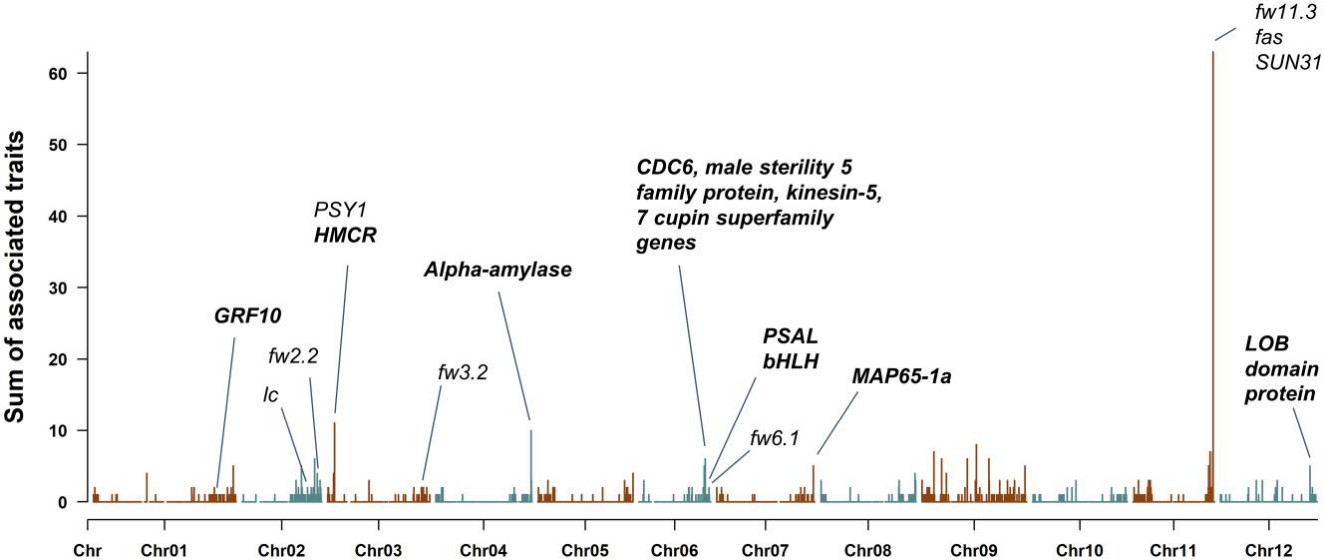
Pan-fruit QTLome—genome-wide cumulative representation of QTL associated with agro-morphological traits. For genome-wide association studies, 445 and 402 *S. lycopersicum* accessions were used for mapping, employing either GBS, which identified 9,536 SNPs (Zemach et al. 2023), or WGS, which detected 1,875,501 SNPs relative to the *S. lycopersicum* 2.5 reference genome (Zemach et al. 2023). Additionally, the GWAS mapping population of Grozeva et al. (2021) was used containing 145 accessions from the Balkans and 22,867 SNPs. In addition, the Brix panel was used employing 7,287 SNP-CHIP (Zemach et al 2023). Two populations were used for linkage mapping, the BIL population derived from *S. pennellii* (LA5240) × *S. lycopersicum* (LEA) (Torgeman and Zamir 2023) and the *recombinant* inbred line population derived from *S. pennellii* (LA0716) × *S. lycopersicum* (cultivar M82; LA3475) (Ofner et al. 2016). Known QTL (*fw2.2*, *fw3.2*, *fw6.1*, *fw11.3*) and genes (*GRF10*, *lc*, *PSY1*, *HMCR*, *alpha-amylase*, *cell division CDC6*, *male sterility 5 family protein*, *kinesin-5*, *cupin superfamily genes*, *photosystem I subunit L* [*PSAL*], *bHLH*, *MAP65-1a*, *fas*, *SUN-family gene 31* [*SUN31*], *LOB domain protein*) are highlighted.

To expand the description of the genetic architecture of tomato fruit morphology and yield-related traits, we integrated whole-genome sequencing (WGS) data from 402 accessions of the 674 tomato accession panel and mapping results from a previously published 145 accession Balkan collection (Grozeva et al. 2021) with data from 3 previously published tomato populations (Ofner et al. 2016; Torgeman and Zamir 2023; Zemach et al. 2023) to generate a genome-wide cumulative representation of QTL associated with agro-morphological traits (Fig. 2). The dataset included 2 genome wide association study (GWAS) panels, one composed of globally sourced accessions using genotyping-by-sequencing (GBS) and WGS data (Zemach et al. 2023) and another focused on accessions from the Balkan region (Grozeva et al 2021), as well as 2 backcross inbred line (BIL) populations derived from crosses between *Solanum pennellii* and *S. lycopersicum*: LA5240 × LEA (Torgeman and Zamir 2023) and LA0716 × M82 (Ofner et al. 2016) (Figs. 2 and  S2 to S7).

We mapped a wide range of fruit-related traits, including fasciation, locule number, fruit color, size, eccentricity, productivity, fruit weight, yield, and others (Data S1). In total, 945 QTL were identified across all populations and analyses (Fig. S2). The majority of QTL were detected in the GWAS 674 panels, with 697 QTL associated with 33 significant traits. In the LEA BIL population, 106 QTL were identified for 12 significant traits, while the Brix panel yielded 102 QTL across 12 traits. The M82 BIL population contributed 72 QTL associated with 50 significant traits, and the GWAS Balkan population revealed 37 QTL linked to 26 traits. Across populations, 133 QTL were shared, indicating partially conserved genetic control. Chromosomes 5, 9, and 10 harbored the highest numbers of QTL, with 100, 196, and 92 loci, respectively, encompassing both previously reported and additional regions. In the following, we highlight 4 known candidate genes alongside 18 additional candidates identified in this study.

The global GWAS panel described by Zemach et al. (2023) successfully recapitulated several known loci, including a well-characterized QTL located at the distal end of Chromosome 11 (Figs. 2 and  S3), encompassing *Solyc11g071840* encoding *SlSUN31* (Tripodi et al. 2021) and the *fas* locus (Cong et al. 2008; Xu et al. 2015). Some QTL and candidate genes comprise fruit color which was mapped to a QTL on Chromosome 6 containing tandem candidate genes *Solyc06g082940* and *Solyc06g082950* (encoding a PSI subunit L) alongside *Solyc06g083170* (a basic helix–loop–helix [bHLH] transcription factor). *Solyc06g082940* and *Solyc06g082950* exhibit high expression at 5 and 20 days postanthesis (dpa) in the pericarp tissue, inner epidermis of mature green and breaker stem, and seeds of *S. lycopersicum* cultivars M82 and SUN1642 (tea.solgenomics.net/expression_viewer; tomexpress.gbfwebtools.fr). The bHLH transcription factor *Solyc06g083170* shows the highest expression at 4 days post anthesis (dpa) in the seed coat of *Solanum pimpinellifolium* (LA1589) and from 10 to 20 dpa in whole seeds and columella of the *S. lycopersicum* M82 cultivar.

In the Balkan GWAS panel, comprising 3 subpopulations (Fig. S4), we identified a QTL associated with the average a* color value (a colorimetric indicator of green-redness), and hue of the fruit. This QTL was mapped to a known locus on chromosome 3 and includes a carotenoid biosynthesis cluster encompassing a *phytoene synthase 1* (*PSY1*, *Solyc03g031860*) (Bartley et al. 1992) and 2 unknown *hydroxy-methylglutaryl-CoA reductase* (*HMCR*) genes (*Solyc03g032010* and *Solyc03g032020*). The 2 *HMCR* genes showed the highest expression at 30 dpa to pink stage in M82 (tea.solgenomics.net/expression_viewer).

From the study by Ofner et al. (2016), 2 yield-related QTL were identified: *Solyc04g082090*, encoding an alpha-amylase, and *Solyc07g064970*, encoding a microtubule-associated protein (MAP65-1a) (Fig. 2; Data S1). The *alpha-amylase* exhibits moderately high expression in the pericarp tissue throughout fruit development, as well as in seeds at 42 dpa and breaker +2 stage in the M82 cultivar (tea.solgenomics.net/expression_viewer, tomexpress.gbfwebtools.fr). *MAP65-1a*, meanwhile, shows strong expression in the septum and columella at 5 dpa and in the pericarp tissue throughout fruit development as well as in seeds from 4 to 20 dpa in M82 (tea.solgenomics.net/expression_viewer, tomexpress.gbfwebtools.fr).

Beyond these morphological descriptors, we investigated fruit weight and identified both known and previously unidentified associations. Known loci such as *fw2.2* were detected (Figs. 2 and  S5a and b), along with a previously unidentified QTL on the distal end of Chromosome 12 (*fw12.1*) in the global GWAS panel of 674 accessions (Zemach et al. 2023). This region includes *Solyc12g010810*, encoding a LATERAL ORGAN BOUNDARIES (LOB) domain protein, previously implicated in plant growth and tomato fruit ripening (Shi et al. 2021). Another robust locus on the bottom of chromosome 6, *fw6.1*, was identified across both GWAS panels and the LEA BIL population from (Torgeman and Zamir 2023) (Figs. 2 and  S5b). This region contains multiple candidate genes with potential roles in fruit development. *Solyc06g076860*, a homolog of control protein 6 (CDC6), showed moderate expression in pericarp tissue 10 dpa in cultivar M82 and at 4 dpa in *S. pimpinellifolium* (LA1589), as well as from 7 to 20 dpa in *S. lycopersicum* (SUN1642 and M82). *Solyc06g075640*, encoding a male sterility 5 family protein, was most strongly expressed in the placenta and columella at 5 dpa in M82 and in the seed coat and funiculus at 4 dpa in *S. pimpinellifolium* (LA1589). Additionally, *Solyc06g075580*, a kinesin-5 involved in cell organization (van der Knaap et al. 2014), was expressed from 5 dpa to the mature green stage in M82 and in seeds from 4 to 10 dpa in both *S. pimpinellifolium* (LA1589) and *S. lycopersicum* cultivar SUN1642 and M82. Seven genes from the cupin superfamily (*Solyc06g075260*, *Solyc06g075270*, *Solyc06g075280*, *Solyc06g075290*, *Solyc06g075300*, *Solyc06g075320*, and *Solyc06g075360*), known regulators of plant development including fruit growth (Hu et al. 2023), were also located within this region. Among them, *Solyc06g075360* showed high expression throughout fruit development in M82 and at 4 dpa in *S. pimpinellifolium* (LA1589; tea.solgenomics.net/expression_viewer, tomexpress.gbfwebtools.fr).

### Identification of a fruit weight QTL: *SlGRF10*

The most intriguing QTL emerged from a GWAS on the previously defined Brix panel of 109 varieties (Zemach et al. 2023) (Fig. S5b), which breaks the negative correlation between fruit weight and Brix (total soluble solids, largely comprising sugars and organic acids). This phenotype-guided selection approach enabled the discovery of a QTL for fruit weight on Chromosome 1, which we designated *fw1.2*, located ∼10 Mb upstream of the known *fw1.1* locus (Pereira et al. 2021). Here, within *fw1.2* QTL, we identified *Solyc01g091540*, encoding *GRF10*, as a causative gene associated with fruit weight.

Analysis of Tajima's *D* and nucleotide diversity in 10 kb windows (Data S2) revealed a region around 85.14 Mb with elevated nucleotide diversity (*π* = 0.001) and intermediate-frequency alleles (Tajima's *D* = −0.7), relative to the genomic background (*π* = 0.0004; D = −1.7). This pattern suggests that multiple haplotypes are maintained at this locus rather than a classic selective sweep. Although *SlGRF10* begins at 85,156,603 bp, the analyzed window (85.14 to 85.15 Mb) lies upstream of the gene. The elevated diversity and allele frequency spectrum in this region are consistent with functional variation in regulatory elements, such as distal promoters or enhancers, that may modulate *SlGRF10* expression. Interestingly, the region also contains an enoyl-CoA hydratase gene (*Solyc01g091520*), which could contribute to metabolic variation in fruit flavor (Ye et al. 2023). Nevertheless, the association between haplotype diversity and fruit weight supports a primary role for regulatory variation affecting *SlGRF10*, while contributions from neighboring genes cannot be excluded.

This association was also detected when mapping fruit weight (Fig. S5a) and inflorescence habit (Fig. S5c) using the GWAS 674 panel (including the Brix panel) with either GBS (9,536 SNPs) or WGS (1,875,501 SNPs) data. Linkage disequilibrium (LD) analysis revealed the WGS-GWAS lead SNP to be in weak LD (*R*^2^ < 0.65) with 4 polymorphic markers located within the coding region of *SlGRF10* (Fig. S5d). Furthermore, a GWAS using 22,867 GBS-derived SNPs from the Balkan population (Grozeva et al. 2021) (Fig. S6) identified a 2-Mb downstream association from *SlGRF10* in linkage equilibrium.

To cross-validate the association at *SlGRF10*, we turned to BILs and their hybrids derived from *S. pennellii* (LA5240) × *S. lycopersicum* (LEA), totaling 1,400 lines (Torgeman and Zamir 2023), where we again detected the QTL and identified a mapping interval of *fw1.2* spanning 85.11 to 85.35 Mb (Fig. S5b). However, this locus was not detected in the 446 BILs and hybrids derived from *S. pennellii* (LA0716) × *S. lycopersicum* (M82) as described by Ofner et al. (2016), potentially due to allelic variation or population-specific effects (Fig. S7).

### Natural variation of *SlGRF10*

**Figure 3:**
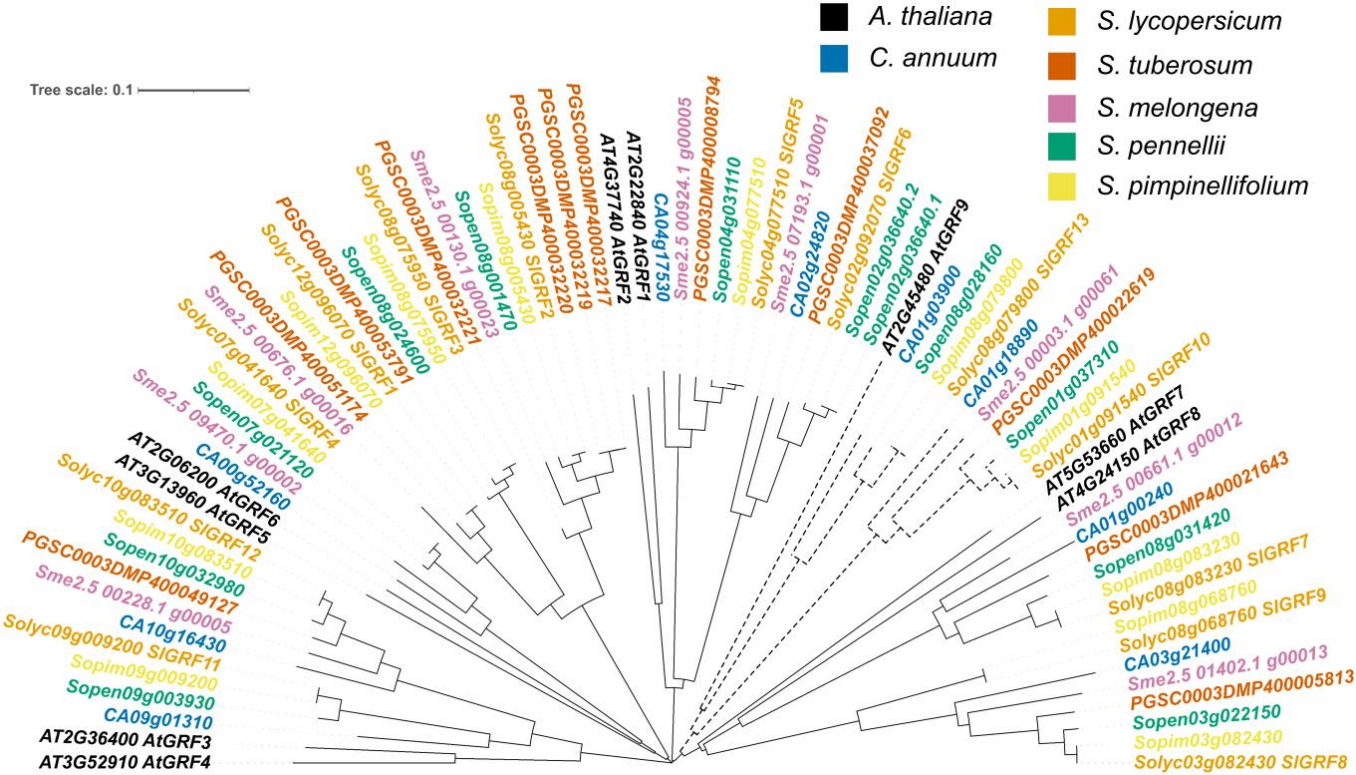
GRF transcription factor family of cultivated tomato, its wild ancestors, eggplant, potato, pepper, and *A. thaliana*. Different colors indicate species and symbols indicate different clades: *S. lycopersicum*, *S. pimpinellifolium*, *S. pennellii*, *S. melongena*, *S. tuberosum*, *C. annuum*, and *A. thaliana*. The clade that includes *SlGRF10* is dotted. Branch lengths of the phylogenetic tree represent genetic distance, and the scale bar indicates the number of substitutions per site.

GRFs are a widespread family of transcription factors in the plant kingdom (Omidbakhshfard et al. 2015). Given the critical importance of fruit size in vegetable crops, we analyzed the GRF gene family across selected wild and cultivated *Solanaceae* species, including *S. lycopersicum*, *S. pimpinellifolium*, *S. pennellii*, *Solanum melongena*, *Solanum tuberosum*, and *Capsicum annuum*, alongside *Arabidopsis thaliana* as an outgroup (Fig. 3). Phylogenetic analysis revealed that orthologues of all *S. lycopersicum* GRFs were present in these species, indicating high conservation of the GRF family. Notably, *SlGRF10* showed strong conservation across species (dotted clade in Fig. 3).

Specific orthologs of *SlGRF10* were identified as follows: *CA01g18890* in pepper, *PGSC0003DMP400022619* in potato, and *Sme2.5_00003.1g00061* in eggplant. Using OrthoFinder, we identified a total of 52,625 orthogroups across the Solanaceae species analyzed (Fig. S8a; Data S3). The orthogroup containing SlGRF10 consisted of 24 orthologs (Fig. S8b). A multiple sequence alignment of these orthologs, including those from wild ancestors and *S. lycopersicum* cv. Heinz, revealed a SNP resulting in an amino acid substitution from alanine to serine at position 311 (Figs. S8c and S9a).

Detailed protein sequence analysis showed that SlGRF10 uniquely possesses 2 WRC motifs and one QLQ motif, distinguishing it from other GRFs (Fig. S9b and c). Structural predictions using AlphaFold confirmed the presence of these 3 highly structured domains, each with a predicted per-residue model confidence score predicted Local Distance Difference Test above 70 (Fig. S9c). The WRC motifs were predicted to be located in β-sheets, while the QLQ motif formed an α-helix. To further understand the functional impact of genetic variation in *SlGRF10*, we performed an in silico analysis of its regulatory and coding sequences. Promoter prediction identified a TATA box located 914 bp upstream of the start codon, with the transcription start site predicted at 882 bp upstream (Data S4).

**Figure 4:**
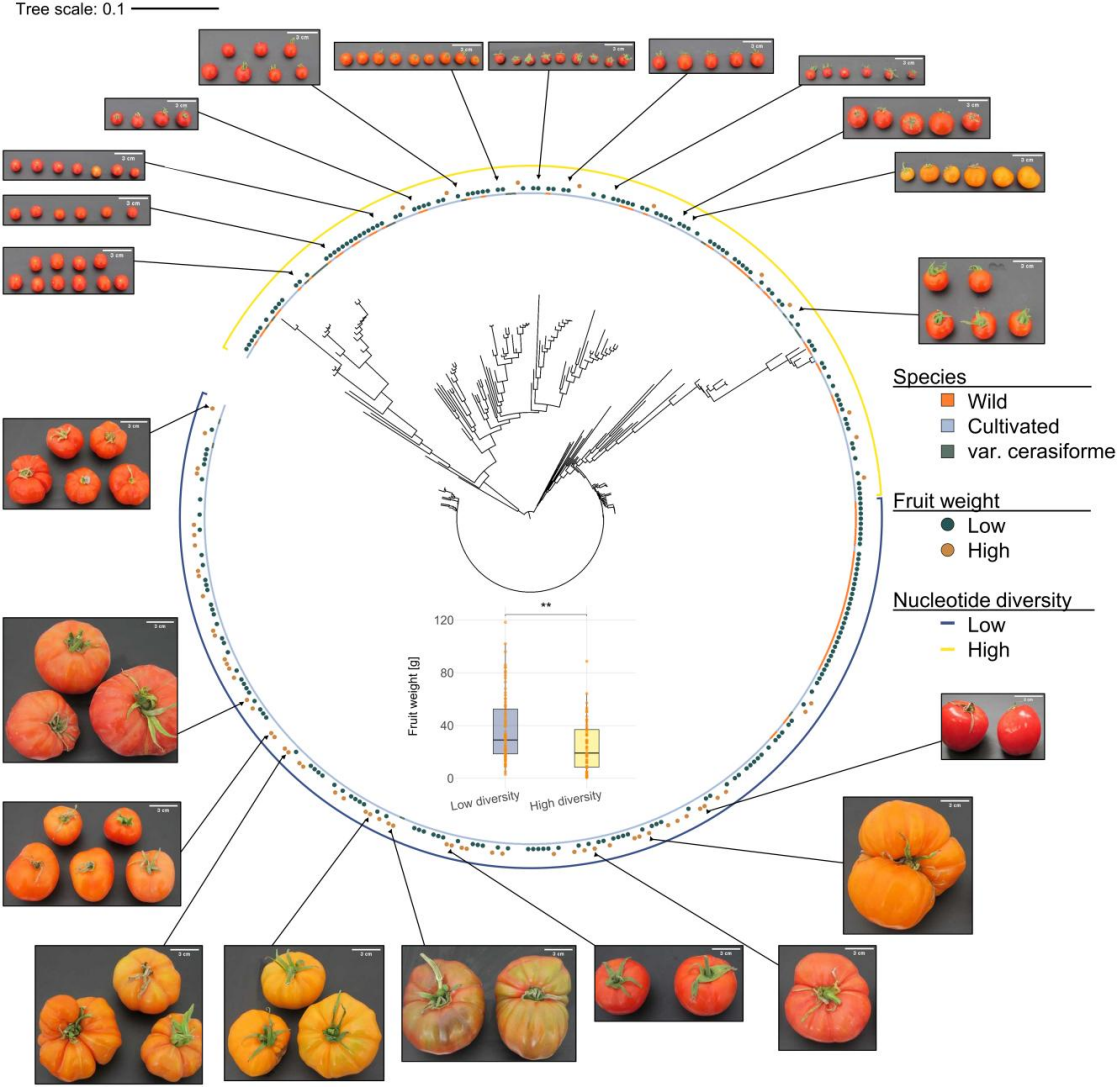
Natural variation of SlGRF10 coding region of 400 polymorphic accessions. Phylogeny contains wild species including *S. cheesmaniae* (2), *S. chilense* (30), *S. chmielewskii* (2), *S. galapagense* (2), *S. habrochaites* (10), *S. huaylasense* (3), *S. lycopersicoides* (1), *S. neorickii* (3), *S. pennellii* (4), *S. peruvianum* (3), *S. pimpinellifolium* (24), cultivated species *S. lycopersicum* (293), and variety *cerasiforme S. lycopersicum* var. *cerasiforme* (23). Dark green circles represent low fruit weight, while dark orange circles high fruit weight (median cutoff). The fruit weight of the accessions with a low nucleotide diversity and high diversity was calculated for cultivated species only (*n*_low diversity_ = 144, *n*_high diversity_ = 71; **P < 0.01, Wilcoxon rank test). Images were obtained from https://unity.phenome-networks.com. Branch lengths of the phylogenetic tree represent genetic distance, and the scale bar indicates the number of substitutions per site.

To investigate natural variation more broadly, we examined SNPs within the coding region of *SlGRF10* using publicly available data from 1,204 tomato accessions (Data S5). After removing redundant or nonunique sequences, 400 polymorphic accessions remained (Fig. 4). We integrated fruit weight measurements and corresponding fruit images for *S. lycopersicum* accessions collected during the field trials reported in Zemach et al. (2023). Grouping the accessions by nucleotide diversity at the SlGRF10 locus (high vs. low diversity defined according to their clustering in the phylogenetic tree) and by fruit weight (low vs. high, based on a median cutoff) revealed that accessions with higher diversity exhibited significantly lower fruit weight compared with those with low diversity.

### Functional validation of SlGRF10

**Figure 5:**
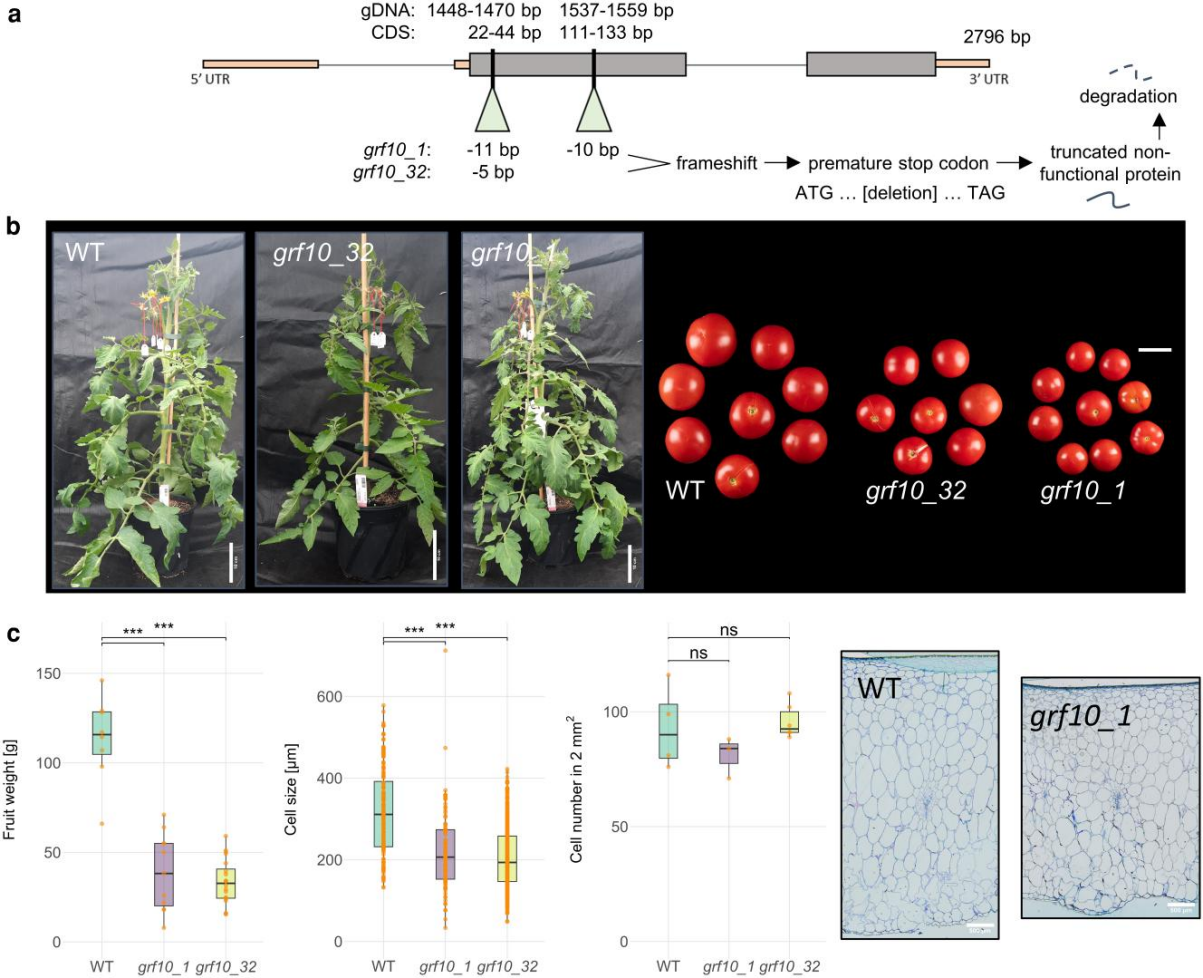
*SlGRF10* CRISPR-Cas9 knockout show decreased fruit weight and cell size. a) Schematic representation of *SlGRF10* with 11 and 5 bp deletion for *grf10_1* and *grf10_32*, respectively, at the target site of sgRNA1 (gDNA: 1,448 to 1,470 bp, coding sequence [CDS]: 22 to 44 bp) and 10 bp deletion at the target site of sgRNA2 (gDNA: 1,537 to 1,559 bp, CDS: 111 to 133 bp). The deletions are predicted to induce a frameshift, leading to a premature stop codon and the formation of a truncated, nonfunctional protein that is likely subject to degradation. b) Representative images of whole plant phenotypes (left) of T_2_ WT (*n* = 5) compared with *grf10_1* (*n* = 5) and *grf10_32* (*n* = 5) after 8 weeks of growth (scale bar = 10 cm) and the representative fruit phenotypes (right) of selected fruits from WT (*n* = 8), *grf10_32* (*n* = 7), and *grf10_1* (*n* = 9) at 17 weeks after pricking in the T_1_ generation (scale bar = 4 cm). Images were digitally extracted for comparison. c) Fruit weight in g (*n*_WT_ = 9, *n*_grf10_1_ = 11, *n*_grf10_32_ = 20), cell number within 2 mm^2^ of pericarp tissue (*n*_WT_ = 4, *n_grf10_1_* = 3, *n_grf10_32_* = 6), and cell size in *µ*m (*n*_WT_ = 160, *n_grf10_1_* = 89, *n_grf10_32_* = 420) of WT compared with *grf10* (left) was recorded of mature red fruits 17 weeks after pricking in the T_2_ generation. Thin-sections (right) of the fruit mesoderm of WT and *grf10_1* (scale bar = 500 *µ*m). Boxplots show median, interquartile range (IQR), and 1.5× IQR whiskers. ***P < 0.001, ns = nonsignificant > 0.05; Kruskal–Wallis test, post hoc Dunn's test.

To investigate the biological role of *SlGRF10*, we explored the function of SlGRF10 by 2 independent CRISPR-Cas9 knockout mutants, *grf10_1* and *grf10_32*. These were generated using 2 single-guide RNAs targeting the first exon (Figs. 5a and  S10). The *grf10_1* line carried 2 homozygous deletions (11 and 10 bp), while *grf10_32* had a single 5 bp homozygous deletion resulting in frame shifts. Consistent with observations in the T_0_ to T_3_ generations (Figs. S10b, S11a, and S13), both mutants exhibited reduced fruit size and weight (Fig. 5c). The magnitude of fruit weight reduction was comparable between CRISPR-Cas9 mutants (38.4%) and natural variants (34.6% relative to accessions carrying low-diversity SlGRF10 haplotypes). However, Brix index was not consistently affected across generations, and no significant changes were detected in plant height or flower morphology (Figs. S10b and S11). Histological analysis of pericarp tissue revealed a significant reduction in cell size in both independent mutant lines, while cell number remained unchanged (Figs. 5c and  S10b and S12).

To uncover downstream effects of *SlGRF10* loss-of-function, we performed a preliminary transcriptome profiling from T_0_ mature green and red fruits (Data S6–S8; Figs S14 and S15). Across all samples, 25,021 genes were detected. Gene expression profiles clearly distinguished red from green fruits (Fig. S14a and b). Principal component analysis (PCA) based on all expressed genes revealed a clear separation between wild type (WT) and the homozygous *grf10_1* mutant, consistent with the heatmap of the most significantly differentially expressed genes (DEGs) (*P*_adj_ <0.05, |log_2_ fold change| >1; Data S6). Notably, the heterozygous *grf10_32* mutant clustered closely with the WT, suggesting functional compensation by the intact allele and dosage sensitivity.

Pairwise comparisons between WT and homozygous *grf10_1* revealed 414 DEGs in mature green fruits (*P*-value <0.05 and |log_2_ fold change| >1; Data S6). Among the DEGs, several transcripts were associated with key biological processes, including cell cycle regulation and genome stability (eg, *Solyc01g067640*, encoding cell division protein kinase 12, CDK12), cell wall biosynthesis (*Solyc03g005450*, encoding a cellulose synthase), and epigenetic and posttranscriptional regulation (eg, *Solyc01g010970* and *Solyc06g074730*, encoding ARGONAUTE 1). Additional DEGs were involved in hormone biosynthesis and signaling, including ethylene-responsive transcription factors (ERFs; eg, *Solyc03g007460*, *Solyc03g026270*, *Solyc08g081650*), as well as meristem maintenance and organogenesis (eg, *Solyc02g086480* and *Solyc11g045530*, encoding LOB domain proteins). Furthermore, several transcription factors and developmental regulators were identified, including multiple MADS-box proteins (eg, *Solyc01g066730*, *Solyc01g092950*, *Solyc04g081000*, *Solyc12g038510*).

To further interpret these transcriptomic changes, we conducted gene ontology (GO) and Kyoto Encyclopedia of Genes and Genomes (KEGG) enrichment analyses using all 1,148 DEGs from mature green pericarp of *grf10_1*, 981 DEGs from red fruits of *grf10_1*, and 663 DEGs from *grf10_32* (Data S8). In green fruits of *grf10_1*, enriched GO terms included integral components of membranes (170 DEGs), responses to abiotic and biotic stimuli (31 immune-related DEGs), positive regulation of transcription (10 DEGs), and nucleic acid-binding transcription factor activity (47 DEGs; Data S8; Fig. S14c). KEGG pathway analysis revealed differential expression of 15 genes in the MAPK signaling pathway and 79 in various metabolic pathways.

In mature red fruits, enriched categories included sequence-specific DNA-binding transcription factors (37 DEGs), nuclear-associated genes (96 DEGs), genes related to cell wall organization and biogenesis (13 DEGs), and metabolic pathways (78 DEGs; Data S8; Fig. S14d). The heterozygous *grf10_32* mutant displayed differential expression of several genes related to the cytoskeleton, cell wall, and metabolism (Fig. S15).

**Figure 6:**
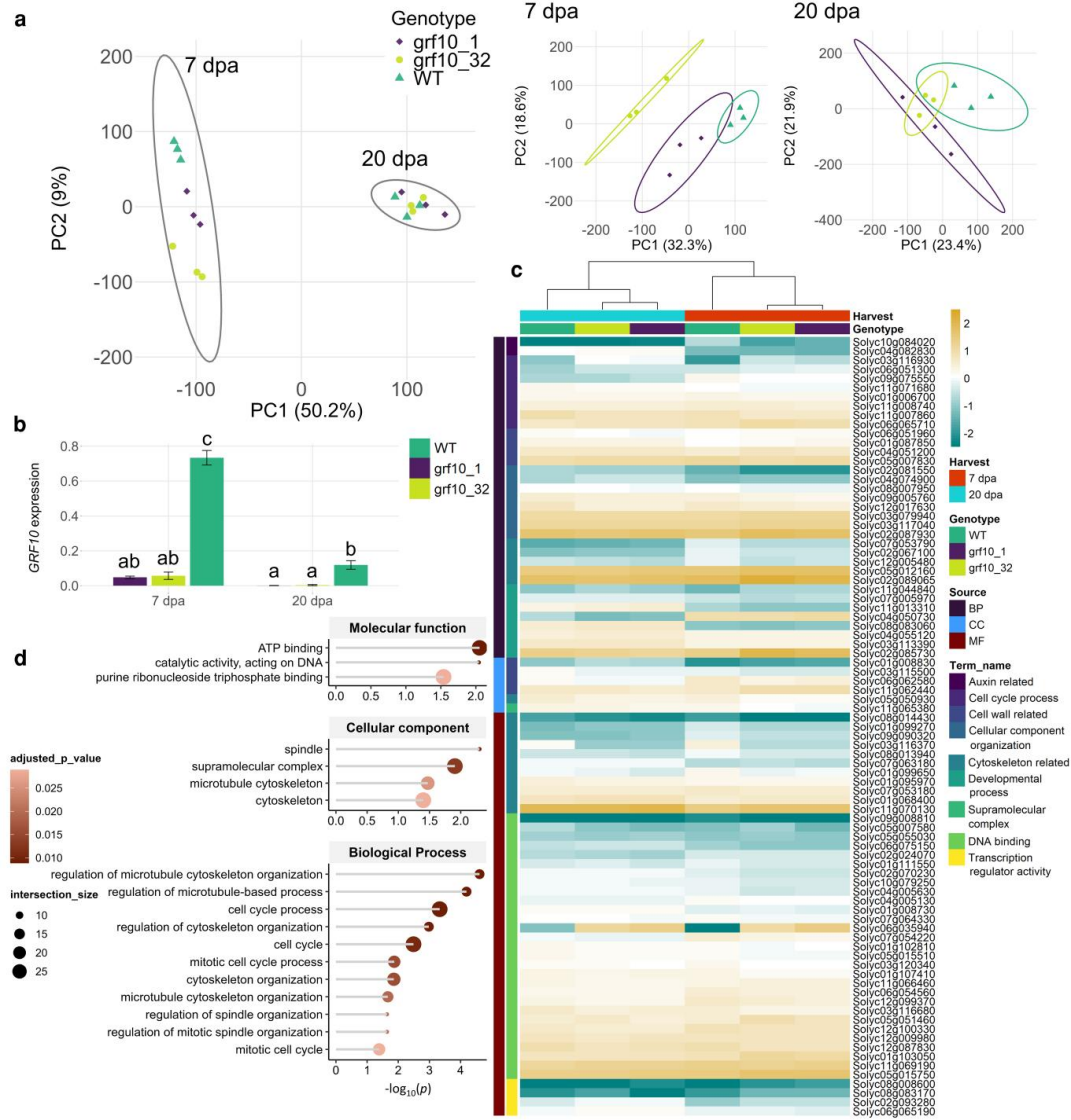
Transcriptomic analysis of *slgrf10* reveals a role in cytoskeleton arrangement and cell cycle control during early fruit development. a) PCA of the fruit pericarp transcriptome of WT and the 2 independent lines *grf10_1* and *grf10_32* harvested 7 and 20 dpa. Samples are shown in a combined PCA including both developmental stages (left) and in separate PCAs for each time point (right). b) Bar plot of *GRF10* expression 7 and 20 dpa in WT, *grf10_1*, and *grf10_32* (*n* = 3, mean ± Se). Letters indicate statistically significant differences (*P* < 0.05) based on 2-way ANOVA followed by Tukey's Honestly Significant Difference (HSD) post hoc test. c) Heatmap of selected DEGs involved in developmental processes base on GO annotation (biological process [BP], cellular component [CC], molecular function [MF]). d) GO enrichment analysis of downregulated DEGs in fruit pericarp comparing WT and *grf10* harvested 7 dpa.

Based on this diverse set of DEGs and to minimize potential confounding effects arising from somaclonal variation in T_0_ plants, we proceeded to analyze the transcriptomes of fruit pericarp samples collected at 7 and 20 dpa from the T_3_ generation, corresponding to peak phases of cell division and cell expansion, respectively. Across all samples, a total of 24,838 genes were detected. PCA clearly separated the 2 developmental stages (Fig. 6a), with stage-specific PCAs revealing a more pronounced genotype-dependent clustering at 7 dpa than at 20 dpa. Differential expression analysis identified 530 DEGs at 7 dpa and 156 DEGs at 20 dpa when comparing WT and *grf10* mutants. In addition, 5,942 and 6,737 DEGs were detected when comparing 7 versus 20 dpa within WT and *grf10*, respectively (*P* < 0.05; Data S9). Consistent with the CRISPR-Cas9-induced mutations, SlGRF10 transcript levels were reduced at 7 and 20 dpa in the mutant and revealed higher *GRF10* transcript abundance at 7 dpa relative to 20 dpa in WT fruit pericarp (Fig. 6b).

Consistent with these results, GO and KEGG enrichment analyses revealed significant enrichment only at 7 dpa (Data S10; Fig. S16a). Specifically, significantly enriched GO terms were detected exclusively among downregulated DEGs and included ATP binding, catalytic activity acting on DNA, cytoskeleton organization, and cell cycle- and cell division-related processes (Fig. 6c and d). The heatmap highlights the expression patterns of representative DEGs associated with these enriched functional categories (Fig. 6c). At 20 dpa, significant enrichment was observed only when up- and downregulated DEGs were analyzed separately: upregulated genes were enriched for terms related to regulation of seedling development and seed germination, whereas downregulated genes were associated with the super elongation complex (Fig. S16b and c).

To facilitate biological interpretation, candidate genes were grouped into 7 functional categories based on GO terms and gene annotation (Data S9). These included the following: (i) cell cycle, chromosome dynamics, and genome integrity, with *Solyc11g071680* and *Solyc08g013940* encoding serine/threonine protein kinases; (ii) cell wall biosynthesis and remodeling, highlighted by *Solyc05g007830*, encoding an expansin; (iii) chromatin remodeling and epigenetic regulation, including *Solyc05g012060* and *Solyc11g011980*, WD-40 repeat proteins previously implicated in grain yield regulation in maize and rice (Chen et al. 2022); (iv) cytoskeleton organization and division plane control, represented by 2 formins (*Solyc08g014430* and *Solyc07g052730*) and 2 microtubule-associated proteins MAP65-1a (*Solyc01g096680* and *Solyc07g064970*); (v) hormone signaling and homeostasis, comprising 10 auxin-related genes, a gibberellin 2β-dioxygenase (*Solyc03g116270*), and a cytokinin oxidase/dehydrogenase (*Solyc08g061930*); (vi) protein turnover, RNA processing, and signaling, including the splicing factor U2AF large subunit (*Solyc09g090420*); and (vii) transcriptional regulation and developmental patterning, encompassing *Solyc12g014400* (cell differentiation protein), 2 MADS-box transcription factors, 2 ERFs, 5 bHLHs, and 3 myeloblastosis (MYB) transcription factors.

Taken together, these results highlight SlGRF10 as a crucial regulator of fruit weight and size in tomato. The observed changes in cellular architecture, gene expression, and phenotypic traits underscore its functional importance. The combination of genome-wide association mapping, natural variation analysis, and functional validation provides a comprehensive view of SlGRF10's role and its potential contribution to domestication and breeding traits.

## Discussion

### Candidate gene identification of morphology QTL

Domestication and modern breeding have shaped the genetic diversity of crops, giving rise to the broad variety of shapes, colors, and sizes observed in cultivated plants today (see Figs. 1 and  S1). As consumer preferences continue to evolve, breeding programs are increasingly targeting quality-related traits that may have been diminished or lost in modern cultivars (Tieman et al. 2017). GWAS provide a powerful approach to rediscover such lost natural variation, enabling the identification of loci that have been genetically depleted during breeding and may hold potential for trait improvement (Tripodi et al. 2021; Goldman et al. 2023). That said, identifying high-yielding varieties and the genetic factors that underlie these traits remain of paramount importance, particularly in light of a growing global population, malnutrition and the pressures of climate change.

In this study, we identified several candidate genes associated with fruit color and yield (Fig. 2; Data S1). These include a QTL associated with color containing *Solyc06g082940* and *Solyc06g082950*, encoding 2 paralogs of PSI subunit L, and *Solyc06g083170*, encoding a bHLH transcription factor. In addition, 2 paralogous *HMCR* genes (*Solyc03g032010* and *Solyc03g032020*) were linked to hue and the a* color value. We also recovered 2 yield-related QTL with the candidates *Solyc04g082090* (encoding an alpha-amylase) and *Solyc07g064970* (encoding the MAP65-1a). Notably, all candidate genes showed expression in tomato fruit tissues, supporting their potential functional relevance in fruit development and quality.

Furthermore, we identified both known and previously unidentified loci associated with fruit morphology and productivity (Figs. S2 to S7). Previously characterized loci, such as *fas* (Cong et al. 2008; Xu et al. 2015), *SlSUN31* (Tripodi et al. 2021), *PSY1* (Bartley et al. 1992), and *fw2.2* (Frary et al. 2000; Chakrabarti et al. 2013), were successfully recovered. We also identified additional candidate genes implicated in fruit weight determination. Among the previously unidentified loci identified, *fw12.1* and *fw6.1* stand out. These QTL harbor a LOB domain gene and a gene cluster involved in cell division, differentiation, and development, respectively, and are reported here. Additionally, we discovered a QTL on chromosome 1 containing *SlGRF10*. Interestingly, this QTL was uniquely detected in the Brix panel and only weakly in the larger, more diverse panel. It was not identified in other GWAS panels, nor in the BIL population derived from *S. pennellii* LA0716 × *S. lycopersicum* M82 (Ofner et al. 2016). However, it was validated in a BIL population derived from the lost accession *S. pennellii* LA5240 in the background of *S. lycopersicum* cv. LEA (Torgeman and Zamir 2023).

These results underscore the importance of using multiple GWAS populations with distinct genetic backgrounds to capture a broader spectrum of QTL. In addition, it also highlights the critical roles of donor parent selection and sufficient sample size in achieving robust and reproducible mapping results.

### SlGRF10 is a key regulator of fruit weight in tomato

GRFs are a conserved family of transcription factors found throughout the plant kingdom, where they govern a wide range of developmental processes across all plant organs (Liu et al. 2023). In rice, for instance, one GRF ortholog controls husk opening and floral identity (Liu et al. 2014). In tomato, all GRF paralogues are strongly expressed in flower buds, with SlGRF10 and its closest paralog SlGRF13 showing the highest expression levels (Khatun et al. 2017).

Tajima's *D* and nucleotide diversity analysis (Data S2) suggest that multiple haplotypes are maintained at the SlGRF10 locus, consistent with balancing selection maintaining functional allelic variation affecting fruit weight, rather than a classic selective sweep. However, the observed correlation between haplotype diversity and fruit weight supports the hypothesis that regulatory variation influencing SlGRF10 plays a key role in determining fruit weight.

To explore the role of SlGRF10 in more detail, we analyzed orthologues in selected crop and ornamental Solanaceae species. This revealed thousands of orthogroups (Data S3), offering a valuable resource for identifying functionally similar genes in related species. For example, given that SlGRF10 influences fruit weight in tomato, *CA01g18890* emerges as a promising candidate for fruit weight studies in pepper (Fig. 3). Structural analysis of *SlGRF10* revealed 2 WRC domains and 1 QX_3_LX_2_Q motif, a unique combination within the *S. lycopersicum* GRF family (Fig. S8). The presence of 2 WRC motifs in *SlGRF10* (Fig. S9) could enhance its regulatory versatility by enabling more robust or selective interactions with growth regulating factor (GIF) cofactors. This structural feature may reflect an evolutionary adaptation to the complex regulation of fruit development and size, potentially offering increased functional redundancy or allowing context-dependent interaction with multiple transcriptional partners.

To investigate natural variation at the *SlGRF10* locus, we analyzed 70 SNPs in the coding region across 1,204 accessions from 12 tomato species. We found that greater genetic diversity was associated with lower fruit weight (Fig. 4; Data S5), suggesting that modern breeding efforts have depleted variation at this locus to favor larger fruits. Supporting this, CRISPR-Cas9 knockout lines of *SlGRF10* produced significantly smaller fruits, and fruit weight loss similar to naturally occurring allelic variation, confirming its role in regulating fruit weight (Figs. 5 and  S10 to S13). In addition, phenotypic and transcriptomic analysis (Figs. S10b and S14) on T_0_ generation suggests a dosage sensitivity which could provide a mechanistic explanation for how naturally occurring weak alleles at the *GRF10* locus contribute to fruit weight variation. The fact that both high genetic diversity and gene knockout result in smaller fruits indicates that SlGRF10 activity may be reduced in small-fruited varieties.

Although the molecular mode of action of GRFs remains only partially understood, emerging models suggest interactions between GRFs, GIFs, MIR396, auxin-responsive factors (ARFs), and other transcription factors to promote growth across diverse tissues (Liu et al. 2023). In tomato, the exact mechanism of GRF activity remains unresolved, as is the case in many other fruit-bearing species. Fruit weight is influenced by cell expansion and division, as well as floral meristem organization and size (van der Knaap et al. 2014). Accordingly, our transcriptomic analysis of fruits at 7 and 20 dpa (Figs. 6 and  S16) focused on genes associated with the cell cycle, cell expansion, hormone signaling, and developmental patterning.

Among the DEGs (Data S9), the cell differentiation protein rcd1 *Solyc12g014400* previously known to maintain meristematic fate in *A. thaliana* (Teotia and Lamb 2011) is likely involved in pericarp tissue patterning, regulation of the transition from cell division to cell expansion, and layer specification of the exocarp, mesocarp, and endocarp. Altered expression of such regulators can lead to changes in pericarp thickness and cell identity, ultimately affecting fruit morphology and weight (Gillaspy et al. 1993; Powell and Lenhard 2012). The cytokinin oxidase/dehydrogenase *Solyc08g061930*, which catalyzes cytokinin degradation, represents another key regulatory node. Reduced expression of cytokinin oxidase/dehydrogenase has been shown to elevate cytokinin levels and prolong the phase of cell proliferation during early fruit development, thereby influencing final fruit size and tissue architecture (Matsuo et al. 2012). Cell expansion–related genes were also affected, including the expansin *Solyc05g007830*. Expansins mediate cell wall loosening and promote cell enlargement, acting downstream of hormones such as auxin and gibberellin. Differential expansin expression can impair proper cell expansion, leading to smaller cells and reduced organ growth (Brummell et al. 1999; Cosgrove 2000). In addition, serine/threonine protein kinases (*Solyc11g071680* and *Solyc08g013940*) were differentially expressed. These kinases, often functioning as cyclin-dependent kinases or MAP kinases, regulate G1/S and G2/M cell cycle transitions, coordinate division plane orientation, and integrate hormonal signals with cell cycle progression (Dewitte and Murray 2003; Inzé and De Veylder 2006). Dysregulation of these pathways may result in prolonged or excessive cell division without proportional cell expansion. ARFs (*Solyc11g069190*, *Solyc01g103050*, *Solyc06g075150*, *Solyc12g042070*, and *Solyc03g121060*) constitute central regulators of auxin-mediated transcriptional responses (De Jong et al. 2009). ARFs control multiple aspects of fruit development, including cell division, cell expansion, tissue polarity, and vascular patterning. Importantly, individual ARFs can exert distinct or even opposing functions, with some promoting cell proliferation and others stimulating cell expansion (Bouzroud et al. 2018).

Despite the reduced mesocarp cell size observed in the mutant (Fig. 5c), GO enrichment analysis at 7 dpa (Fig. 6d; Data S10) revealed a strong overrepresentation of cell cycle and cytoskeleton-related genes. This pattern suggests prolonged or misregulated cell proliferation, likely delaying the transition from cell division to cell expansion. Such a proliferation–expansion tradeoff has been widely reported during plant organ growth (Gonzalez et al. 2012) and is consistent with the established role of GRFs in promoting cell division (Omidbakhshfard et al. 2015).

Overall, these findings support the concept that fruit size and shape are not determined solely by cell expansion, but rather emerge from the precise temporal coordination of cell division, differentiation, and expansion.

The tomato clade has undergone intensive breeding for increased fruit size and yield, making it an ideal model to study fruit weight—a complex trait governed by multiple pleiotropic genes (van der Knaap et al. 2014). Our findings demonstrate that reduced genetic diversity at the *SlGRF10* locus correlates with increased fruit size, while increased diversity may compromise the protein's function, leading to smaller fruits. This is supported by our knockout mutant data. Overall, this study not only validates the role of SlGRF10 in controlling fruit weight but also provides a rich resource of candidate genes and DEGs relevant to morphological and agronomical traits. The identification of orthologues in other Solanaceae species, such as eggplant and pepper, further broadens the impact and translational potential of our findings.

## Conclusion

In this study, we combined GWAS, gene expression profiling, mutant analysis, and cross-species comparison with identify and functionally validate SlGRF10 as a regulator of fruit weight in tomato. Our findings demonstrate that SlGRF10 affects fruit weight predominantly through changes in mesocarp cell size rather than cell number, implicating it in cell expansion processes. Transcriptome analysis of CRISPR-Cas9 knockout lines further revealed widespread changes in genes associated with growth, development, transcriptional regulation, and hormone signaling, consistent with the proposed function of GRF transcription factors. Notably, the natural variation analysis suggests that *SlGRF10* has undergone selective pressure during domestication, with reduced allelic diversity correlating with increased fruit size. This not only validates the importance of SlGRF10 in shaping agronomically relevant traits but also highlights the utility of diverse mapping populations in uncovering genetic factors that may be invisible in conventional breeding panels. Our findings contribute to the growing understanding of the molecular mechanisms regulating fruit development and provide a valuable resource of candidate genes for targeted breeding in tomato and related Solanaceae crops.

## Material and methods

### GWAS and linkage analysis

For GWAS, following populations were used: a globally sourced GWAS panel of 674 accessions using GBS (Zemach et al. 2023) and WGS (Tieman et al. 2017) data, a Brix GWAS panel (Zemach et al. 2023), and a Balkan GWAS population comprising 145 accessions (Grozeva et al. 2021). The R package rMVP (Yin et al. 2021) was used employing efficient mixed-model association as variance component method to correct for population structure effects with a kinship matrix and 3 principal components (PCs) as fixed effects. Mixed linear models was used as an association testing method for the identification of significant associations between genetic variants (SNPs) and the phenotype of interest. We used the Bonferroni correction to adjust the significance threshold for multiple comparisons. Candidate genes were identified by scanning the genetic area ± 100 kb of the lead SNP. For QTL/linkage mapping 2 inbred populations were used: a BIL population deriving from *S. pennellii* (LA5240) × *S. lycopersicum* (LEA) (Torgeman and Zamir 2023) and a recombinant inbred line population derived from *S. pennellii* (LA0716) × *S. lycopersicum* (cultivar M82; LA3475) (Ofner et al. 2016). R/qtl (Broman et al. 2003) was used to construct a linkage map using marker regression (method = “mr”) as a linear regression method of the single QTL model. The threshold was further defined by a permutation rate of 1,000 and an alpha threshold of 0.01. For clean visualization, the logarithm of odds threshold was defined as 15.

### Tajima's *D* and nucleotide diversity

The variant call format (VCF) file of chromosome 1 was processed using VCFtools v0.1.16 (Danecek et al. 2011). We retained only biallelic SNPs, removed indels, and excluded sites with missing genotype calls in more than 20% of individuals and with minor allele count <2. The resulting filtered VCF file was used for calculation of nucleotide diversity and Tajima's *D* in a 10-kb window.

### Phylogenetic analysis

OrthoFinder v2.0.0 (Emms and Kelly 2015, 2019) was used to search for orthologs in *A. thaliana* (TAIR10), *S. lycopersicum* (ITAG4.0), *Nicotiana benthamiana* (Niben261), *Nicotiana attenuata* (NIATTr2), *Nicotiana tabacum* (Nitab-v4.5), *C. annuum* (Pepper.v.1.55), *Coffea humblotiana* (cohum), *Iochroma cyaneum* (IC_v1.0), *Petunia axillaris* (v.1.6.2), *Petunia inflata* (v1.0.1), *S. tuberosum* (PGSC_DM_v.3.4), *S. melongena* (Sme2.5), *S. lycopersicum* var. *cerasiforme* (SLYcer_r1.1), *Solanum lycopersicoides* (SlydLA2951_v2.0), *Solanum chilense*, *S. pennellii* (Spenn-v2), and *S. pimpinellifolium* (SPI_r1.1). Protein sequences were obtained from The Arabidopsis Information Resource (TAIR) (https://www.arabidopsis.org/) and Solgenomics (https://www.solgenomics.net/). Sequences of the GRF transcription factor family from *A. thaliana*, *S. lycopersicum, S. tuberosum*, *S. melongena, S. pennellii*, *S. pimpinellifolium,* and *C. annuum* were obtained from http://planttfdb.gao-lab.org/family.php?sp=Sly&fam=GRF. Trees were visualized using iTOL (https://itol.embl.de/). For multiple sequence analysis, Clustal Omega (https://www.ebi.ac.uk/Tools/msa/clustalo/) and alignment viewer (https://alignmentviewer.org/) were used.

### Protein structure prediction

AlphaFold prediction was carried out using DeepMind's Colab notebook (https://colab.research.google.com/github/deepmind/alphafold/blob/main/notebooks/AlphaFold.ipynb#scrollTo=lUQAn5LYC5n4), and the protein structure was visualized using the Research Collaboratory for Structural Bioinformatics Protein Data Bank (https://www.rcsb.org/3d-view) (Berman et al. 2000).

### In silico promoter prediction

For the promoter prediction of *GRF10*, the nucleotide sequence 1.5 kb upstream of the start codon was used as input for Transcription Start Site program (TSSP) analysis as described in (Solovyev et al. 2010).

### Natural variation of *SlGRF10*

SNPs in the tomato genome were identified for 1,204 accessions, comprising 957 *S. lycopersicum*, 135 *S. lycopersicum* var. *cerasiforme*, 2 *Solanum cheesmaniae*, 34 *S. chilense*, 3 *Solanum chmielewskii*, 2 *Solanum galapagense*, 11 *Solanum habrochaites*, 3 *Solanum huaylasense*, 1 *S. lycopersicoides*, 3 *Solanum neorickii*, 4 *S. pennellii*, 3 *Solanum peruvianum*, and 46 *S. pimpinellifolium* (Data S5). Raw sequencing reads were downloaded from NCBI Sequencing Read Archive and processed to remove adaptor and low-quality bases using Trimmomatic (v0.39) (Bolger et al. 2014). Cleaned reads were aligned to the tomato reference genome (SL4.0) (Hosmani et al. 2019) using BWA-MEM (v.0.7.17-r1188) (Li, 2013) with default parameters. Duplicate read pairs were marked, and variants were called using the Sentieon package (https://www.sentieon.com/). SNPs were filtered using GATK (McKenna et al. 2010) with parameters “QD < 2.0 || FS > 60.0 || MQ < 40.0 || MQRankSum < −12.5 || ReadPosRankSum < −8.0”. SNPs within the genomic region of *SlGRF10* were subsequently extracted for further analysis.

### CRISPR-Cas9 mutant construction

Cloning was performed following Reem and Van Eck (2019) as described in Wijesingha Ahchige et al. (2023). In brief, 2 steps of Golden Gate cloning are used to assemble a level 2 vector, carrying 2 single-guide RNAs (target side sgRNA1: 1,448 to 1,470 bp genomic DNA [gDNA], 22 to 44 coding [cDNA]; target side sgRNA2: 1,537 to 1,559 bp gDNA, 111 to 133 bp cDNA; designed using ChopChop https://chopchop.cbu.uib.no/), a kanamycin selection marker and the Cas9 endonuclease. Tomato calli were transformed with the final construct using *Agrobacterium tumefaciens* strain GV2260-mediated transformation by the green team of the MPIMP. Primer sequences used for cloning are provided in Data S11. DNA was extracted following Berendzen et al. (2005). Positive *S. lycopersicum* cv. MoneyMaker transformants were selected by amplification of DNA flanking the target sites and restriction enzyme-mediated identification. PCR fragments of positive lines were purified and sequenced by Sanger sequencing at LGC Genomics (Berlin, Germany). For the T1 generation, only homozygous transformants without the Cas9 transgene were selected.

### Tomato growth conditions

Plants were grown in the greenhouse under long-day conditions as described in Wijesingha Ahchige et al. (2023). In brief, potentially transformed plants were cultivated in a tissue culture chamber under sterile conditions (York International/Johnson Controls, Cork, Ireland; ∼35 *µ*mol m^−2^ s^−1^ of light in a 16 h/8 h day/night cycle at 22 °C). Seeds of positive transformants were obtained and sowed for segregation on soil in a Phytotron growth chamber (York International/Johnson Controls; 150 *µ*mol m^−2^ s^−1^ of light in a 16 h/8 h—22 °C/18 °C—70% relative humidity (RH)/70% RH day/night cycle). Seedlings were transferred into individual pots in greenhouse chambers (16 h/8 h—22 °C/20 °C—50% RH/50% RH—day/night cycle) and grown until fruit maturity.

### Phenotyping

For the T_0_ generation, 3 vegetative clones derived from tissue culture were grown in the greenhouse. For the T_1_ to T_3_ generations, 5 biological replicates per line were cultivated. Fruit weight was recorded for individual fruits in the T_0_ to T_2_ generations, whereas in the T_3_ generation, multiple fruits per plant were weighed together and the mean value was used for analysis. Soluble solid content (Brix) was measured for each fruit using a digital refractometer (ORF 3SM, Kern Optics). Plant height was measured from the soil–air interface to the shoot apical meristem.

### Transcriptome analysis—mature green and red ripe fruits

Three biological replicates of tomato WT, homozygous *grf10_1* and heterozygous *grf10_32* in T_0_ generation fruit were harvested at the mature green and red ripe stages, cut in half and seeds and locular gel removed. The remaining pericarp tissues were flash frozen in liquid N_2_ and ground to a powder for storage at −80 °C. RNA was extracted as described (Chang et al. 1993). Strand-specific RNA-Seq libraries were constructed as per (Zhong et al. 2011), and sequencing was performed on a NextSeq500 instrument (Illumina) at Psomagen, Inc. (Rockville, MD, USA).

Raw RNA-Seq reads were processed to remove adaptors and low-quality sequences using Trimmomatic (version 0.39) and to remove poly-A/T tails using PRINSEQ++ (v1.2) (Cantu et al. 2019). The remaining cleaned reads were aligned to the ribosomal RNA database (Quast et al. 2013) using bowtie (version 1.1.2) (Langmead 2010) allowing up to 3 mismatches, and those aligned were discarded. The final cleaned reads were aligned to the tomato reference genome (SL4.0) using HISAT2 (version 2.1.0) (Kim et al. 2019) with default parameters. Based on the alignments, raw read counts for each gene were calculated and then normalized to fragments per kilobase of transcript per million mapped reads (FPKM). Raw read counts were then fed to DESeq2 (Love et al. 2014) to identify DEGs using a cutoff of adjusted *P*-value <0.05 and |log_2_ fold change| ≥1.

### Transcriptomic analysis—7 and 20 dpa

The fruit pericarp tissues of 3 biological replicates of WT, *grf10_1*, and *grf10_32* (T_3_ generation) fruits were harvested at 7 and 20 dpa. Flowers were labeled following manual pollination using flower vibration. Total RNA was extracted from the harvested tissues using the NucleoSpin RNA Plant Kit (Macherey-Nagel). RNA sequencing was performed by Novogene (Munich, Germany). Total RNA quality was assessed prior to library preparation. Polyadenylated RNA was enriched from total RNA using poly-T oligo-attached magnetic beads, fragmented, and reverse-transcribed using random hexamer primers. Second-strand cDNA synthesis was performed using dUTP to generate strand-specific libraries. Libraries were constructed following standard Illumina protocols, including end repair, A-tailing, adapter ligation, size selection, PCR amplification, and purification. Library quality and concentration were assessed using Qubit fluorometry, quantitative PCR, and an Agilent Bioanalyzer. Sequencing was performed on an Illumina NovaSeq X Plus platform using paired-end sequencing (PE150). Raw reads were processed to remove adapter contamination, reads containing more than 10% ambiguous nucleotides, and reads with low base quality, as previously described (Yan et al. 2013). Clean reads were aligned to the reference genome using HISAT2, a graph-based splice-aware aligner optimized for RNA-seq data (Mortazavi et al. 2008). Transcript assembly and transcript identification were performed using Cufflinks (Pertea et al. 2015). Gene expression levels were quantified based on mapped reads and normalized as FPKM to account for sequencing depth and gene length (Mortazavi et al. 2008; Trapnell et al. 2010; Bray et al. 2016). Differential expression analysis was performed on log_2_-transformed FPKM values using gene-wise *t*-tests or Wilcoxon tests based on the data normal distribution followed by Benjamini–Hochberg correction for multiple testing.

### Gene enrichment analysis

GO is a widely used bioinformatics tool for characterizing gene product functions across species using defined GO terms (Ashburner et al. 2000). GO annotation provides a powerful framework for predicting gene functions and understanding biological processes. GO terms and KEGG pathways enriched in DEGs were identified using a cutoff of *P*-value <0.05 and |log_2_ fold change| >1. Functional enrichment analysis of significant genes was performed using g:Profiler (https://biit.cs.ut.ee/gprofiler/gost).

### Histological analysis

Tomato pericarp samples of the T_0_, T_2_, and T_3_ generations were cut into 5 mm transverse segments and fixed in Formalin Acetic acid Alcohol solution (45% ethanol, 5% glacial acetic acid, 5% formaldehyde [37%], in distilled water) for 24 h. Samples were dehydrated through an ethanol series (50%, 70%, 80%, 90%, 100%) and stained in 0.1% Eosin Y (in 90% ethanol) overnight at 4 °C. Preinfiltration was performed in a 1:1 mixture of 100% ethanol and Technovit 7100 (Kulzer), followed by infiltration in Solution A (Technovit 7100 + 1% Hardener I). Embedding was done in Solution B (15:1 of Solution A to Hardener II) and polymerized overnight at room temperature. Sections (5 μm) were cut using a Leica RM2265 microtome, mounted in distilled water, and dried at 42 °C. Slides were stained with 0.05% Toluidine Blue O, rinsed, dried, and imaged using an Olympus BX51 microscope in bright-field mode. Image analysis was performed using Fiji (ImageJ). Cell number was determined by counting individual cells within a 2 × 2 mm region located between the cuticle and the vasculature. Cell size was assessed by measuring the length of cells along a transect from the cuticle toward the mesophyll within the same region.

### Data analysis

Plots were designed using the R version 4.3.2 and the R package “ggplot2.” Heatmap was created using “Pheatmap.” Pan-fruit QTLome plot was created using the package “CMplot.” Significances were computed with either Student's *t*-test or Wilcoxon rank test or for multiple testing, ANOVA or Kruskal–Wallis test based on the data normal distribution.

## Accession numbers

Sequence data from this article can be found in the GenBank/EMBL and Sol Genomics Network databases. The accession number of the major gene characterized in this study is SlGRF10 (Solyc01g091540). Detailed information on genes, guide RNAs, and CRISPR-Cas9 constructs used in this study is provided in Data S11.

## Supplementary Material

kiag465_Supplementary_Data

## Data Availability

All data generated and analyzed in this study are presented in this article and in the additional files.
