# Supplementary material for: Natural variation in SlGRF10 reveals a role in regulating tomato fruit weight
Source: Plant Physiol. 2026 Jul 1;201(3):kiag465. doi: 10.1093/plphys/kiag465 (PMC13360278; doi:10.1093/plphys/kiag465)
Supplement: kiag465_Supplementary_Data [file kiag465_supplementary_data.zip › GRF10_SI_figures_editorial.pdf]

Supplementary Figures

Natural variation of *SlGRF10* reveals a role in tomato fruit weight

Julia von Steimker[1], Markéta Macho[1], Regina Wendenburg[1], Jeongah Lee[1], Itay Zemach[2], Yimin Xu,[3] Anja Fröhlich[1], Arun Sampathkumar[1], Dani Zamir[2], Zhangjun Fei[3], James J. Giovannoni[3,4], Alisdair R. Fernie[1,5], Saleh Alseekh[1,5]*

[1] Max-Planck-Institute of Molecular Plant Physiology, Am Mühlenberg 1, 14476, Potsdam-Golm, Germany

[2] Hebrew University of Jerusalem, Robert H. Smith Institute of Plant Sciences and Genetics in Agriculture, Rehovot, Israel.

[3] Boyce Thompson Institute for Plant Research, Cornell University, Ithaca, New York, USA.

[4] US Department of Agriculture/Agriculture Research Service, Robert W. Holley Centre for Agriculture and Health, Ithaca, New York, USA.

[5] Center of Plant Systems Biology and Biotechnology, 4000 Plovdiv, Bulgaria

*Correspondence (alseekh@mpimp-golm.mpg.de).

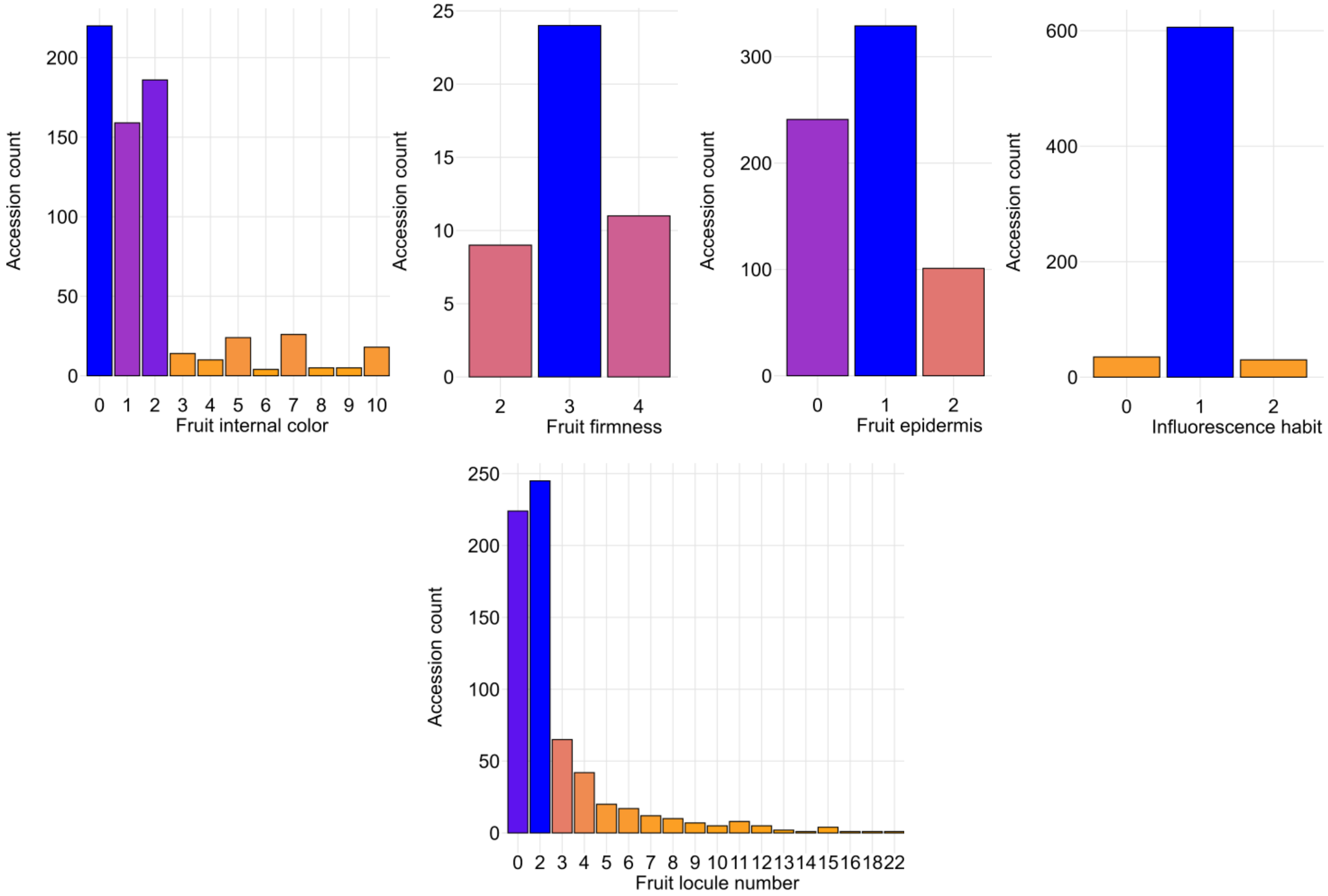

Supplementary Figure S1. Morphological descriptors of 674 selected tomato accessions. Bar plots of fruit internal color (0, white/colorless; 1-2, very pale; 3-4, light; 5-6, intermediate; 7-8, dark; 9-10, very dark), fruit firmness (2, soft; 3, medium; 4, firm), fruit epidermis (0, thin; 1, intermediate; 2, pronounced), influorescence habit (0, simple/unbranched; 1, intermediate; 2, compound/highly branched), and the locule number. The accessions include cultivated *S. lycopersicum*, *S. lycopersicum* var. *cerasiforme,* and wild species. Updated based on Zemach et al. (2023). Color scale represents trait values, ranging from low (orange) to high (blue).

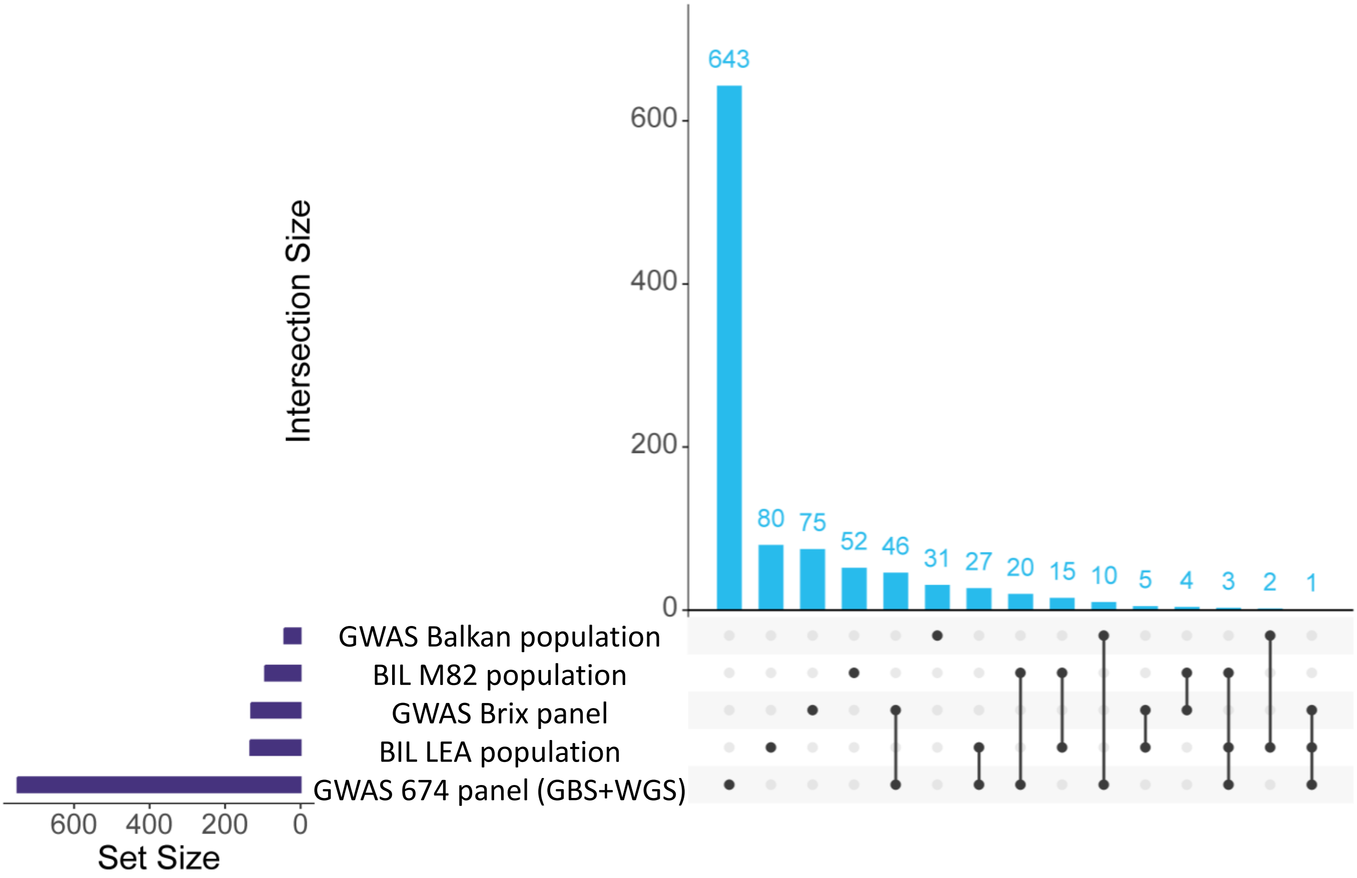

Supplementary Figure S2. Quantitative trait locus (QTL) summary of GWAS and BIL populations used in this study. Common QTL identified in a 50 kb sliding window through mapping of agro-morphological traits in the GWAS Balkan population (Grozeva et al., 2021), backcross inbred line (BIL) M82 population (Ofner et al., 2016), GWAS Brix panel and 674 panel (Zemach et al., 2023) using genotyping-by-sequencing (GBS) and whole-genome sequencing (WGS) data, and the BIL LEA population (Torgenman and Zamir, 2023). UpSet plot showing the overlap of QTL identified across five mapping populations. The upper bar plot ("Intersection Size") indicates the number of QTL shared among the population combinations shown directly below. The y-axis represents the number of QTL within each intersection. In the lower matrix, each row corresponds to one mapping population and each column represents a specific combination of populations. Black dots indicate populations included in a given intersection, whereas gray dots indicate populations not included. Vertical lines connect black dots to highlight the populations contributing to each intersection. The horizontal bar plot on the left ("Set Size") shows the total number of QTL identified in each individual population, irrespective of overlap with other populations. Together, the plot summarizes both population-specific QTL and QTL shared among multiple mapping populations.

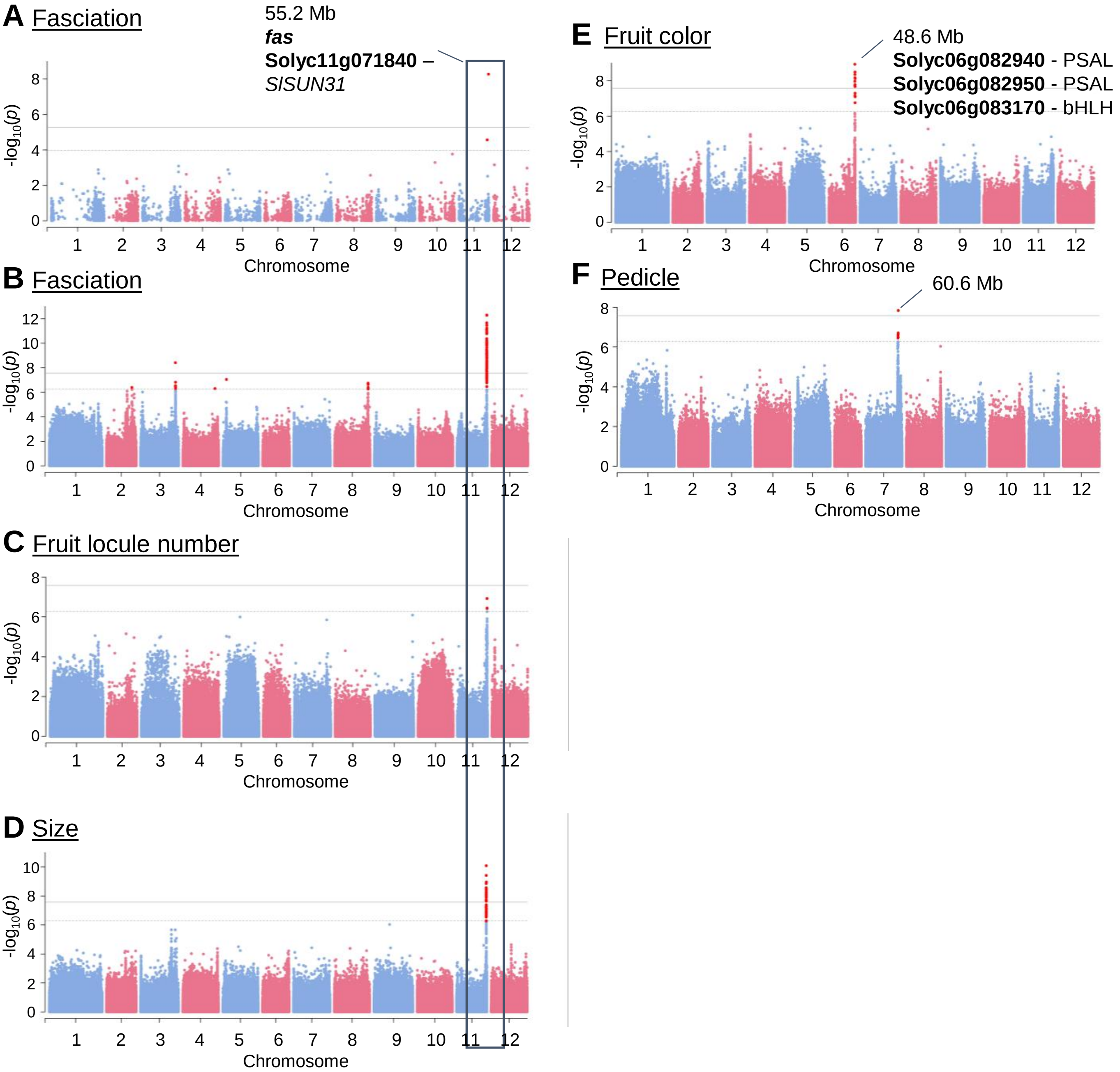

Supplementary Figure S3. Genome-wide association study (GWAS) of morphological descriptors of the 500 globally sourced panel using GBS and WGS data. A) GWAS of fasciation of 445 genotypes using genotyping by sequencing (GBS) data and uncovering 9,536 single-nucleotide polymorphisms (SNPs) and of **B)** fasciation, **C)** fruit locule number, **D)** size, **E)** fruit color and **F)** pedicle shape of 402 *Solanum lycopersicum* accessions using whole-genome sequencing (WGS) calling 1,875,501 SNPs against the *S. lycopersicum* 2.5 genome. Mb = Megabases, PSAL = photosystem I subunit L, bHLH = basic helix-loop-helix. Updated based on by Zemach et al. (2023). Chromosomes are displayed in alternating colors to facilitate visual separation of adjacent chromosomes; colors have no biological significance. Significant single-nucleotide polymorphisms are highlighted in red

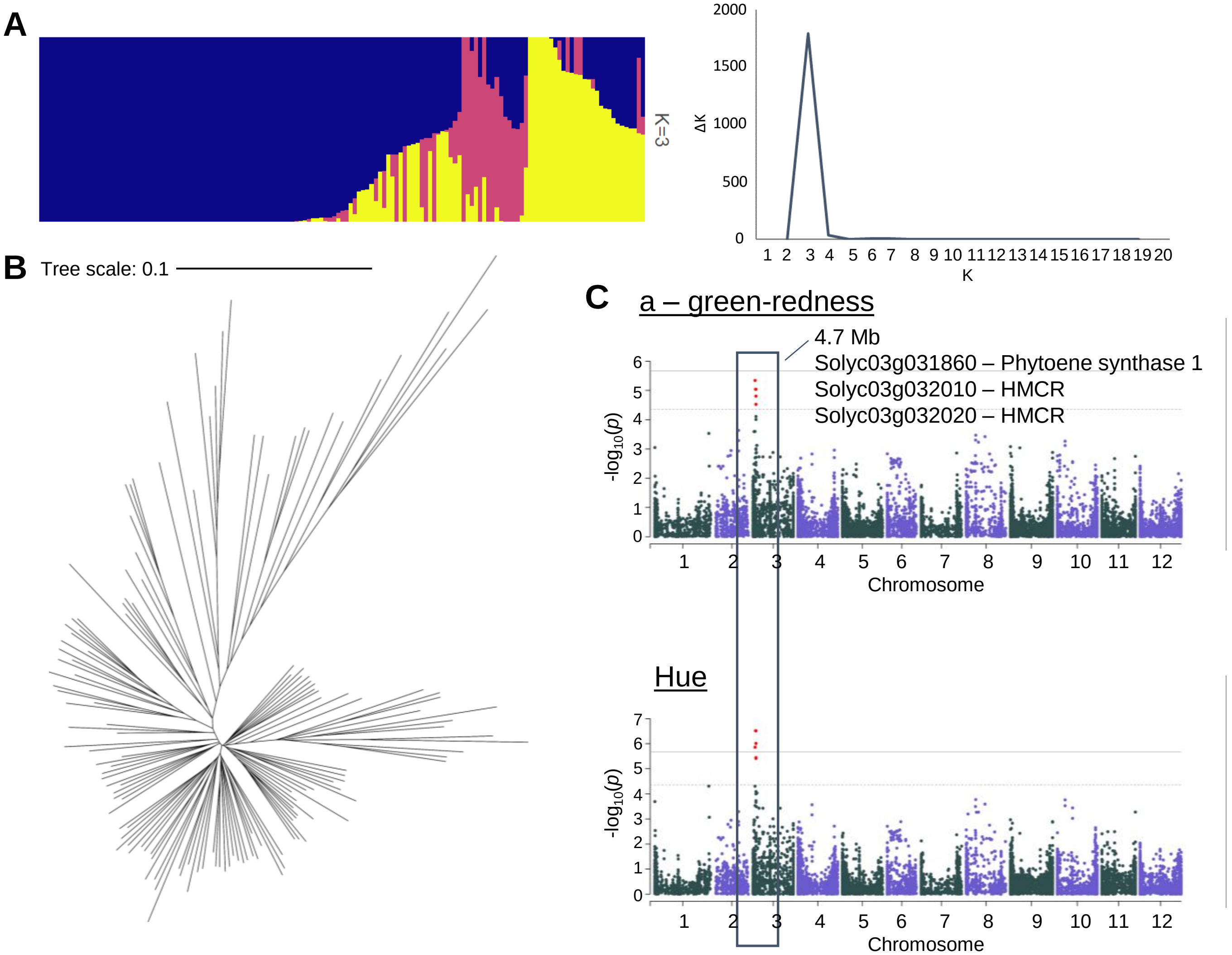

Supplementary Figure S4. Genome-wide association study of morphological descriptors of 145 *S. lycopersicum* accessions from the Balkans. A) Hierarchical population structure analysis with Delta K plot showing the number of estimate populations and **B)** phylogenetic tree of 145 accessions of *S. lycopersicum* from the Balkans. Using 22,867 single nucleotide polymorphisms (SNPs), the number of subpopulations was most likely inferred at K = 3. Population structure at K = 3 based on Q matrix sorted by cluster. Colors represent subpopulations. Branch lengths of phylogenetic tree represent genetic distance, and the scale bar indicates the number of substitutions per site. **C)** Manhattan plots of fruit a-value (green-redness) and hue using 22,867 genotyping-by-sequencing single nucleotide polymorphisms. Mb = megabases, HMCR = Hydroxy-methylglutaryl-coenzyme A reductase. Chromosomes are displayed in alternating colors to facilitate visual separation of adjacent chromosomes; colors have no biological significance. Significant single-nucleotide polymorphisms are highlighted in red

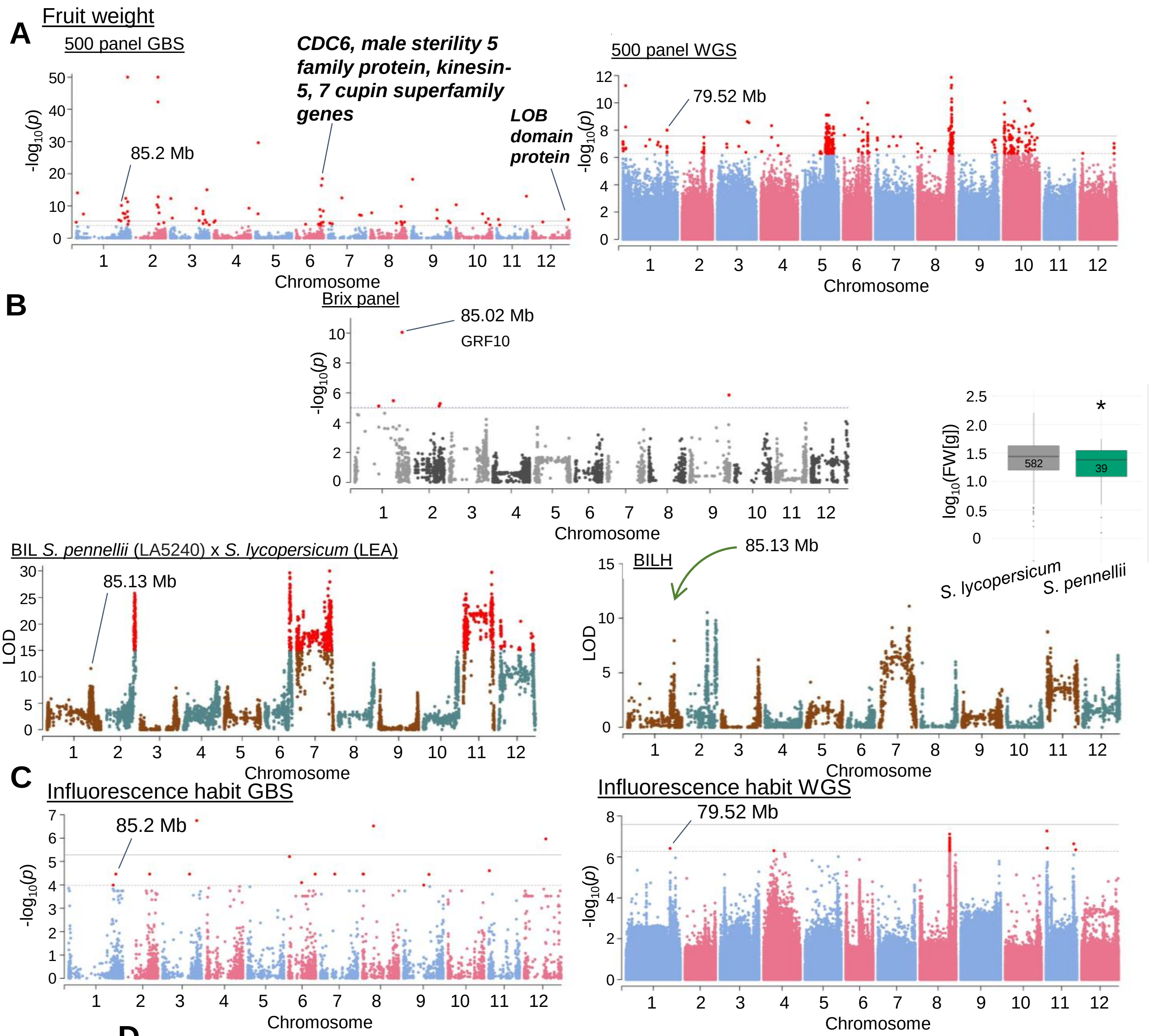

D

Position 1 [bp] WGS lead SNP	Position 2 [bp] WGS GRF10 SNP	States	Distance [bp]	R^2	DPrime	pDiseq	N
79,410,271	85,157,904	A:G	5,628,587	0.596	0.847	2.86E-22	374
79,410,271	85,158,709	A:G	5,629,392	0.641	0.898	4.85E-24	367
79,410,271	85,158,790	G:A	5,629,473	0.58	0.839	5.34E-21	365
79,410,271	85,158,956	T:C	5,629,639	0.584	0.853	9.97E-23	370

Supplementary Figure S5. Identification and cross-validation of GRF10 QTL. A) Genome-wide association study (GWAS) Manhattan plots (purple/pink) of fruit weight (FW) of 445 and 402 *Solanum lycopersicum* accessions using the genotyping by sequencing (GBS) approach discovering 9,536 single-nucleotide polymorphisms (SNPs) or whole-genome sequencing (WGS) calling 1,875,501 SNPs against the *S. lycopersicum* 2.5 genome (Zemach et al., 2023). **B)** GWAS of Brix panel (dark/light grey) and QTL mapping (brown/turquoise) of fruit weight of a backcross inbred line (BIL) population and their hybrids (BILH) from *S. pennellii* (lost accession, LA5240) and *S. lycopersicum* (LEA) published in Torgeman & Zamir 2023. Boxplot shows $\log_{10}$ median, interquartile range (IQR), and 1.5x IQR whiskers of FW in gramm, *p<0.05 (Student's *t*-test), $n_{S.\ lycopersicum} = 582$, $n_{S.\ pennellii} = 39$. **C)** Manhattan plots of the influorenscence habit using either GBS or WGS data of the GWAS 445 and 402 tomato accessions (purple/pink). **D)** Linkage disequilibrium was calculated from the WGS data for the lead SNP against all sites (R^2, correlation; Dprime, historical recombination; pDiseq, p-value for disequilibrium; N, individuals). GWAS thresholds indicate Bonferroni (solid) and false-discovery rate (dotted), for QTL mapping an LOD of 15 was set as threshold. CDC6, cell division control 6; GRF10, growth regulating factor 10; Mb, megabases; bp, base pairs; LOD, logarithm of odds. Chromosomes are displayed in alternating colors to facilitate visual separation of adjacent chromosomes; colors have no biological significance. Significant single-nucleotide polymorphisms are highlighted in red

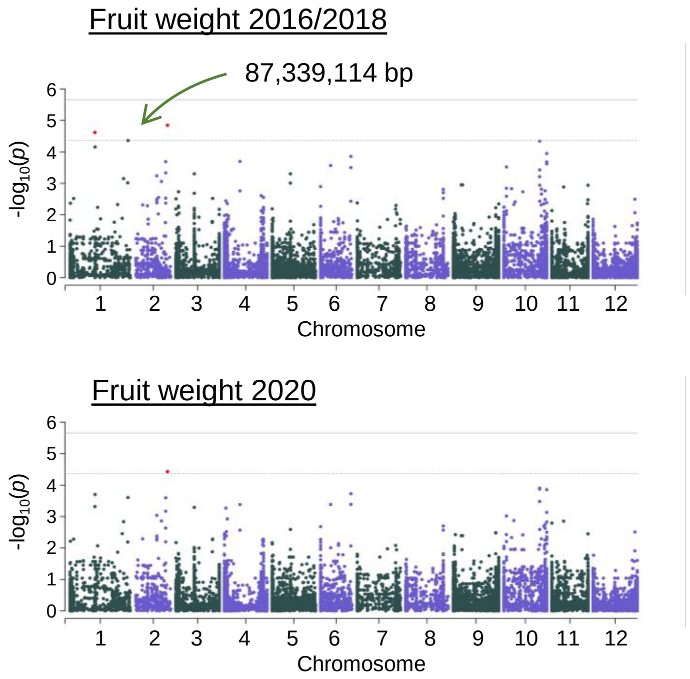

Supplementary Figure S6. A 145 genome-wide association study (GWAS) panel from the Balkans does not validate *SlGRF10*. GWAS results as Manhattan plots of the averaged fruit weight from the years 2016/2018 and 2020. The arrow indicates the lead SNP at 87.3 Mb which is 2 megabases downstream of *SlGRF10* in linkage equilibrium. Chromosomes are displayed in alternating colors to facilitate visual separation of adjacent chromosomes; colors have no biological significance. Significant single-nucleotide polymorphisms are highlighted in red

446 BILs from *S. pennellii* (LA0716) x *S. lycopersicum* (M82) published in Ofner et al., 2016

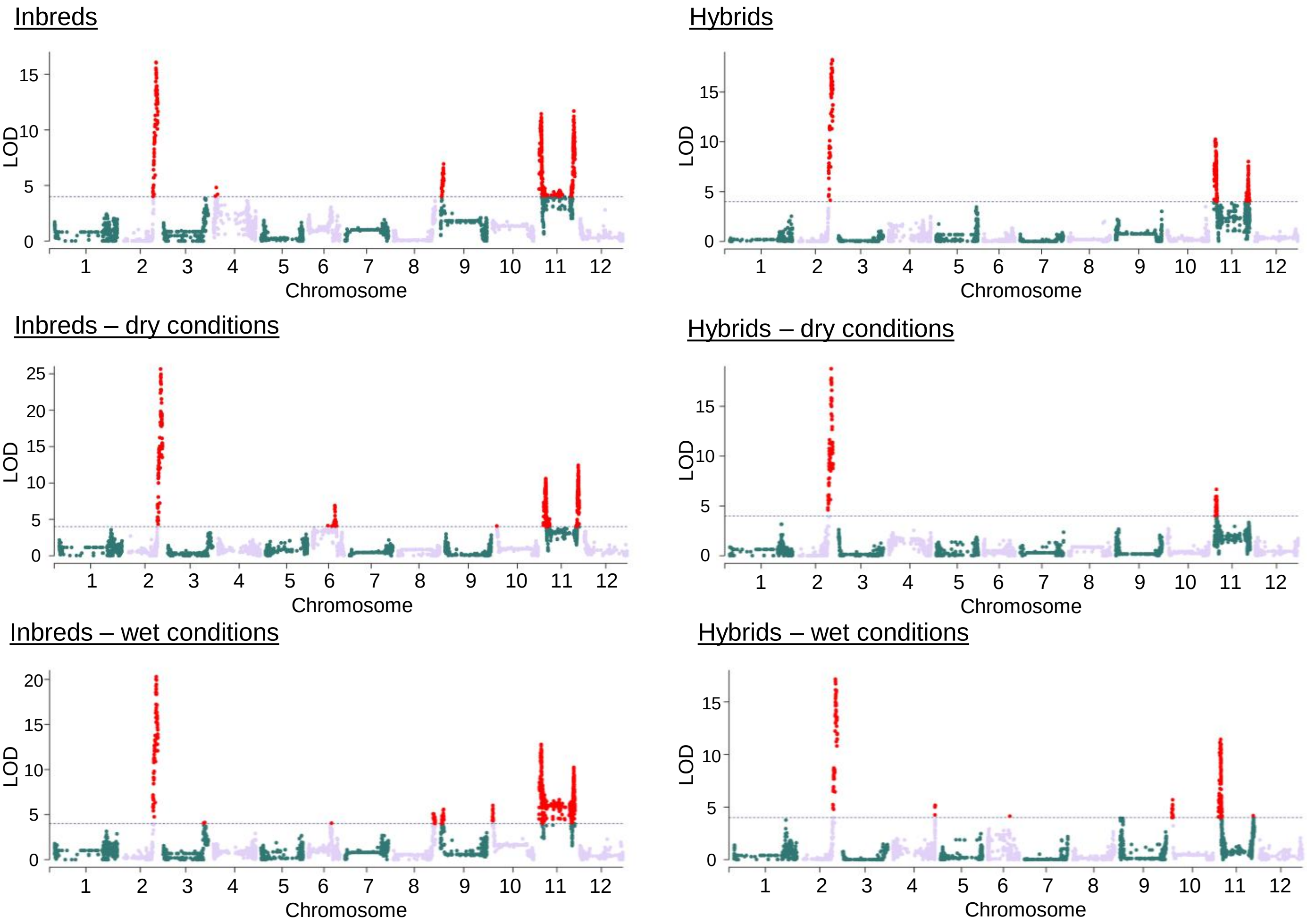

Supplementary Figure S7. Cross-validation of SlGRF10 using backcross inbred line populations from *Solanum lycopersicum* and *S. pennellii*. QTL mapping of fruit weight of a BIL population and their hybrids of Ofner et al, 2016 using *S. pennellii* LA0716 and cultivated *S. lycopersicum* cv. M82. Only the BIL population from Torgeman and Zamir (2023) containing 1400 BILs could cross validate the QTL of *SlGRF10*. Chromosomes are displayed in alternating colors (rose/turquoise) to facilitate visual separation of adjacent chromosomes; colors have no biological significance. Significant single-nucleotide polymorphisms are highlighted in red. LOD, logarithm of odds.

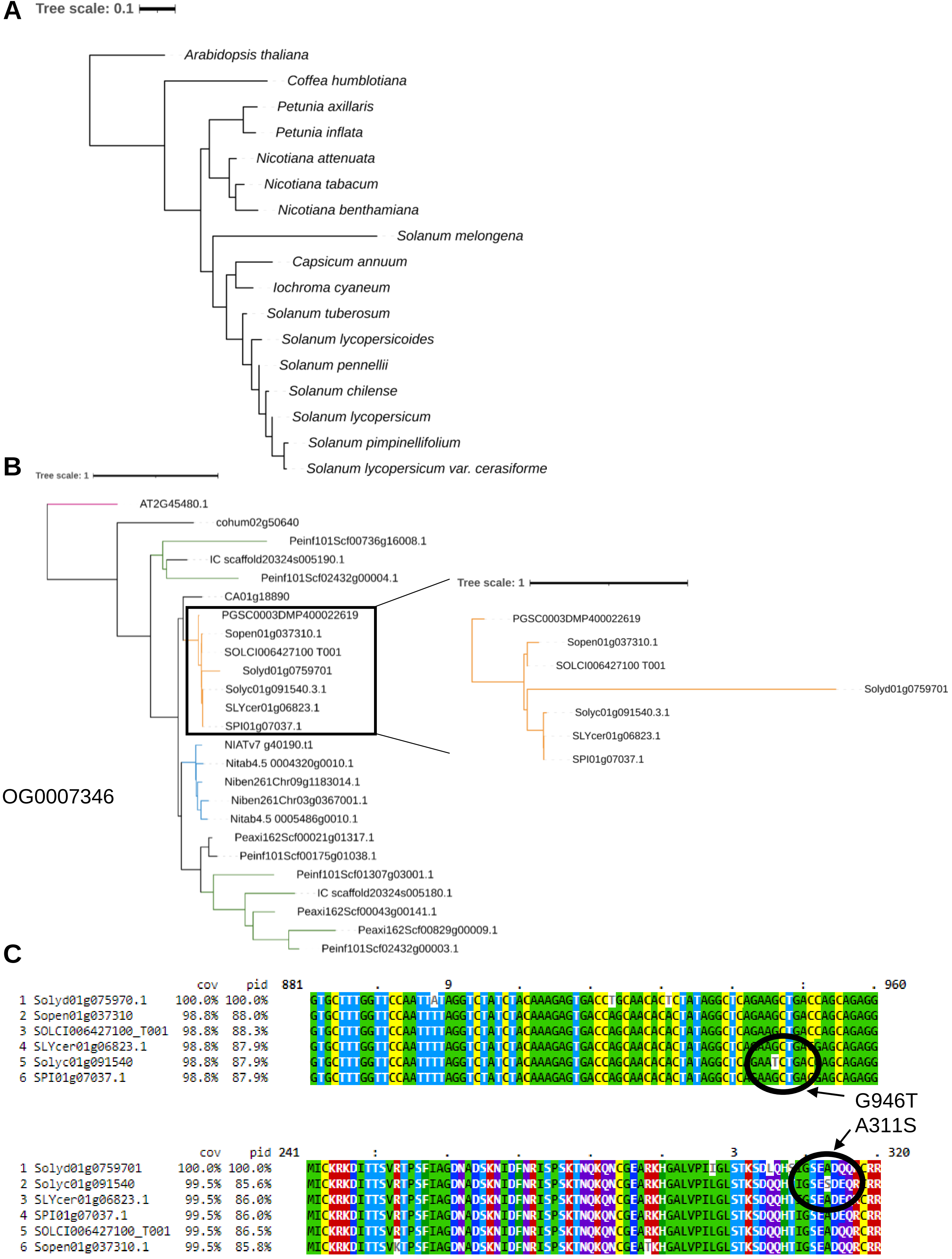

Supplementary Figure S8. An orthologous gene search of *SlGRF10*. A) By searching for orthologous across Solanaceae species including wild tomato species 28,158 orthogroups could be detected. **B)** The gene tree of orthogroup OG007346 with zoom into the tomato clade and **C)** multiple sequence analysis identified the genes of *Solanum pimpinellifolium* and *S. lycopersicum* var. *cerasiforme* as being the closest as well as uncovering an amino acid substitution (G946T, A313S). Branch lengths of phylogenetic trees represent genetic distance, and the scale bar indicates the number of substitutions per site.

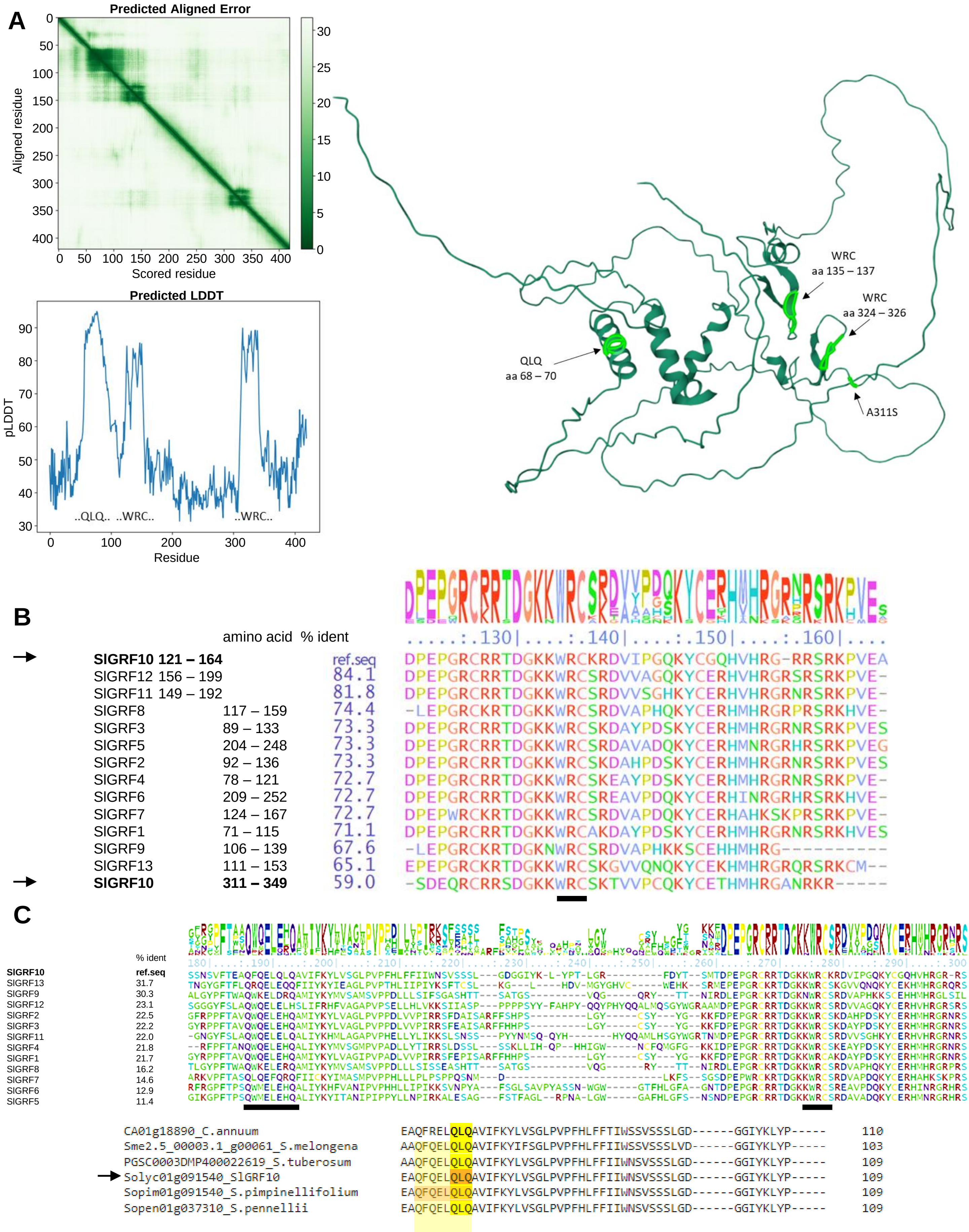

Supplementary Figure S9. SlGRF10 possesses a QLQ and two WRC motifs. A) AlphaFold predicted the three motifs with a per-residue model confidence score (pLDDT) higher 70 and an error rate of less than 5. The tertiary protein structure with an emphasis on the QLQ, and both WRC motifs together with the alanine to serine substitution from Figure S8. **B)** Multiple sequence alignment (MSA) of the growth-regulating factor (GRF) transcription factor family in tomato with a focus on the WRC motif and the **C)** QLQ (QX_3LX_2Q) motif highlighted in yellow with *SlGRF10* QLQ in orange. The arrows indicate SlGRF10.

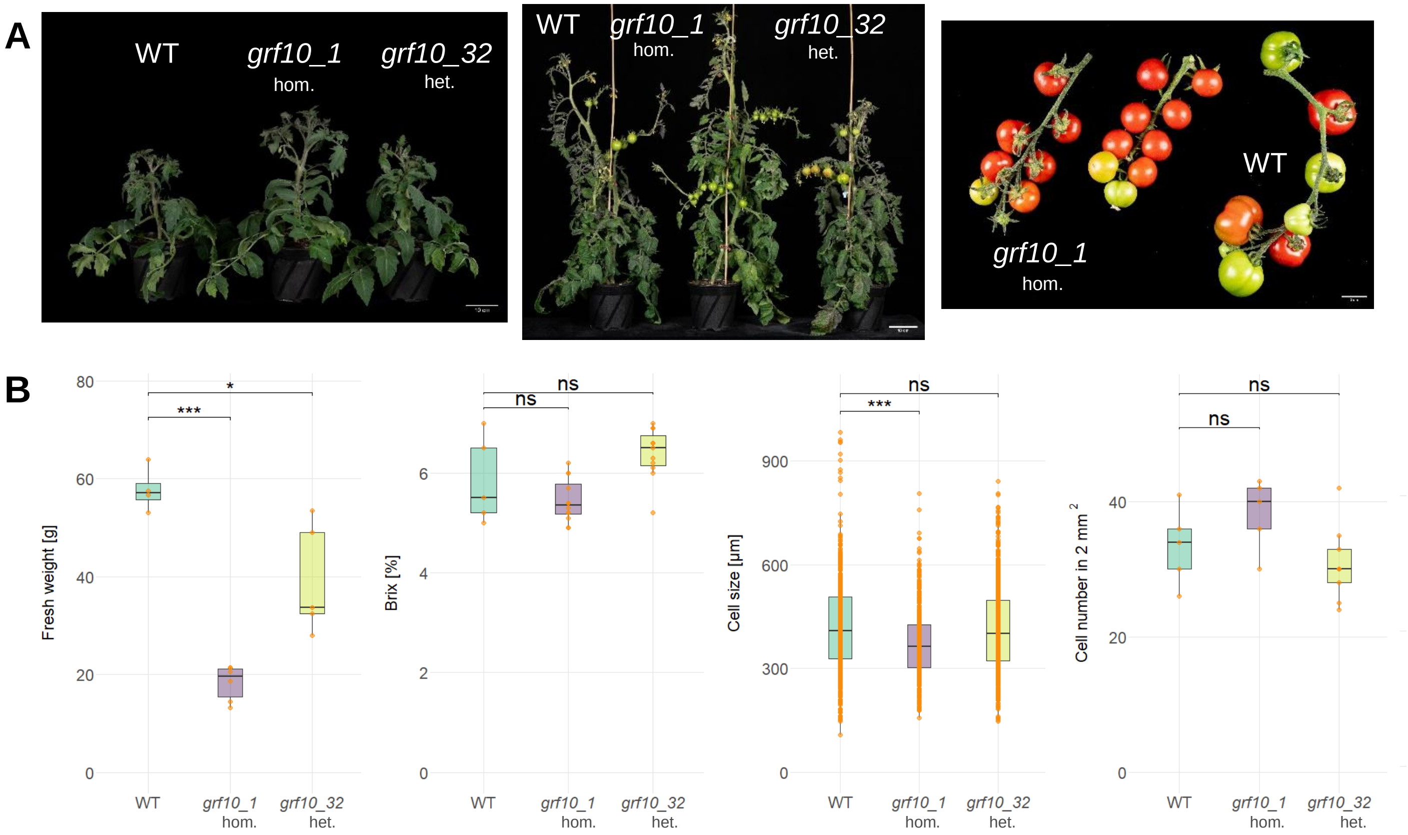

Supplementary Figure S10. T_0 SlGRF10 CRISPR-Cas9 knock-out showed a decreased fruit weight and cell size and no change in Brix and cell number. A) The phenotype of wild type (WT) compared to homozygous (hom.) *grf10_1* and heterozygous (het.) *grf10_32* after seven weeks and 12 weeks after pricking and of the fruits 17 weeks after pricking. Images were digitally extracted for comparison. **B)** Fruit weight in g (n_{WT} = 4, n_{grf10_1} = 6, n_{grf10_32} = 5), Brix in % (n_{WT} = 5, n_{grf10_1} = 12, n_{grf10_32} = 11), cell size in µm (n_{WT} = 574, n_{grf10_1} = 400, n_{grf10_32} = 495), and cell number within 2 mm^2 of pericarp tissue (n_{WT} = 5, n_{grf10_1} = 5, n_{grf10_32} = 9) was recorded 17 weeks after pricking (*$p < 0.05$, ***$p < 0.001$, ns = non-significant > 0.05; fruit weight, Brix, cell number using Student's *t*-test, cell size using Wilcoxon rank test). Boxplots show median, interquartile range (IQR), and 1.5x IQR whiskers.

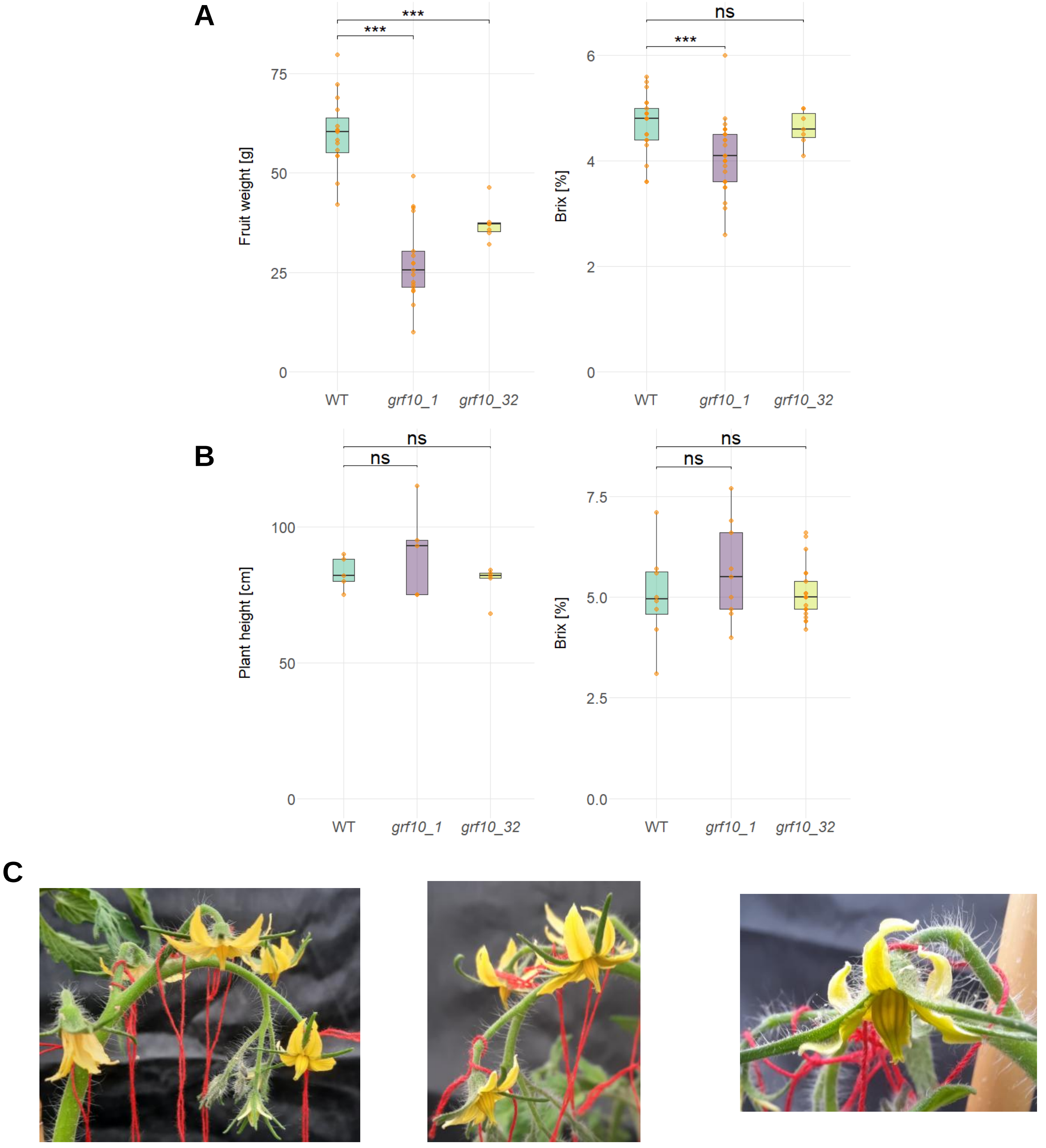

Supplementary Figure S11. T_1 and T_2 SlGRF10 CRISPR-Cas9 knock-out showed a decreased fresh weight, Brix level and no change in plant height. A) T_1 fruit weight in g (n_{WT} = 16, n_{grf10_1} = 15, n_{grf10_32} = 9) and Brix in % (n_{WT} = 21, n_{grf10_1} = 29, n_{grf10_32} = 7). **B)** Plant height of T_2 wild type (WT) and *grf10* mutants (*grf10_1* and *grf10_32*) in cm (n_{WT} = 5, n_{grf10_1} = 5, n_{grf10_32} = 5) after 8 weeks of growth and fruit Brix in % (n_{WT} = 9, n_{grf10_1} = 11, n_{grf10_32} = 20) after 17 weeks of growth. Boxplots show median, interquartile range (IQR), and 1.5x IQR whiskers. **C)** Flower phenotype of 8 weeks old plants. ***p < 0.001, ns = non-significant > 0.05; Student's *t*-test.

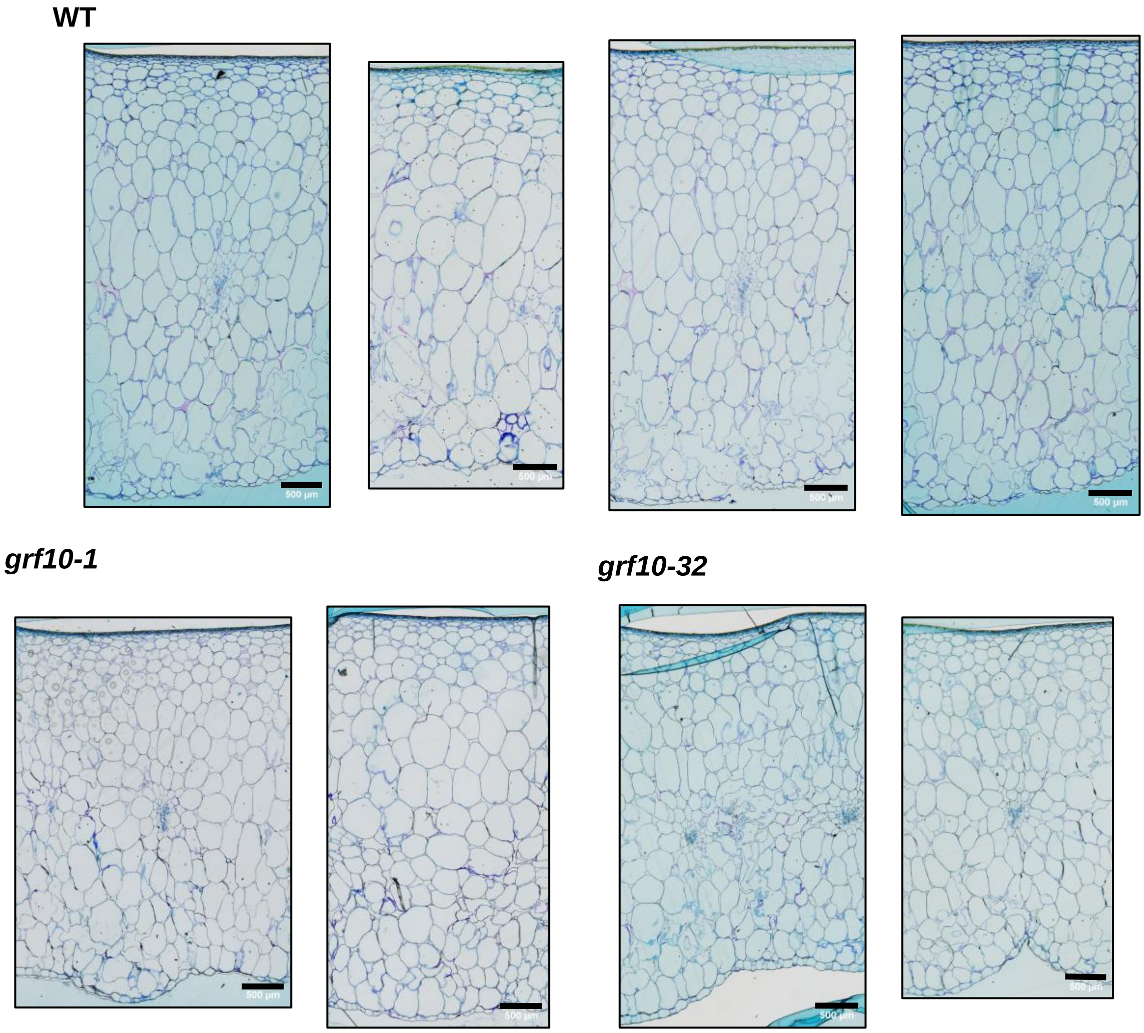

Supplementary Figure S12. Thin sections of T_2 SlGRF10 CRISPR-Cas9 knock-out pericarp show decreased cell size for *grf10*. Thin section of wild-type (WT) *Solanum lycopersicum* cv. MoneyMaker and *grf10*. Sections were 5 µm thick and stained with 0.05 % toluidine blue. The first image of the rows are the same as for Figure 5C. Scale bar = 500 µm.

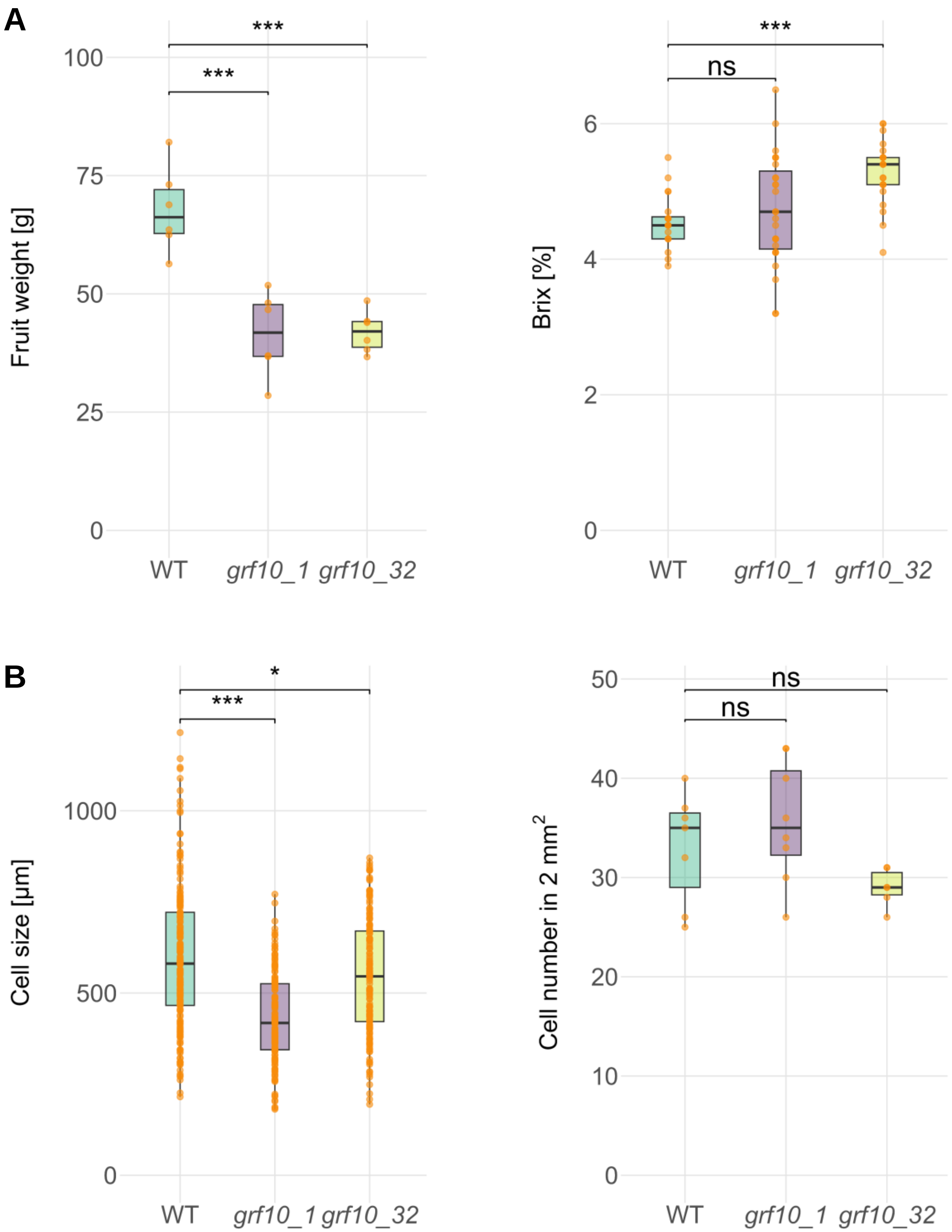

Supplementary Figure S13. Fruit weight, brix, cell size and number of T$_3$ *grf10*. A) Fruit weight [g] (n = 5, average per plant across multiple fruits) and brix [%] (n_{WT} = 20, n_{grf10_1} = 27, n_{grf10_32} = 24) of wild type (WT), *grf10_1* and *grf10_32*. **B)** Cell size [µm] (n_{WT} = 188, n_{grf10_1} = 152, n_{grf10_32} = 147) and cell number in a 2 mm^2 square (n_{WT} = 7, n_{grf10_1} = 8, n_{grf10_32} = 6) based on thin-sections (*p < 0.05, ***p < 0.001, ns = non-significant > 0.05; fruit weight, Brix, cell number using Student's *t*-test, cell size using Wilcoxon rank test). Boxplots show median, interquartile range (IQR), and 1.5x IQR whiskers.

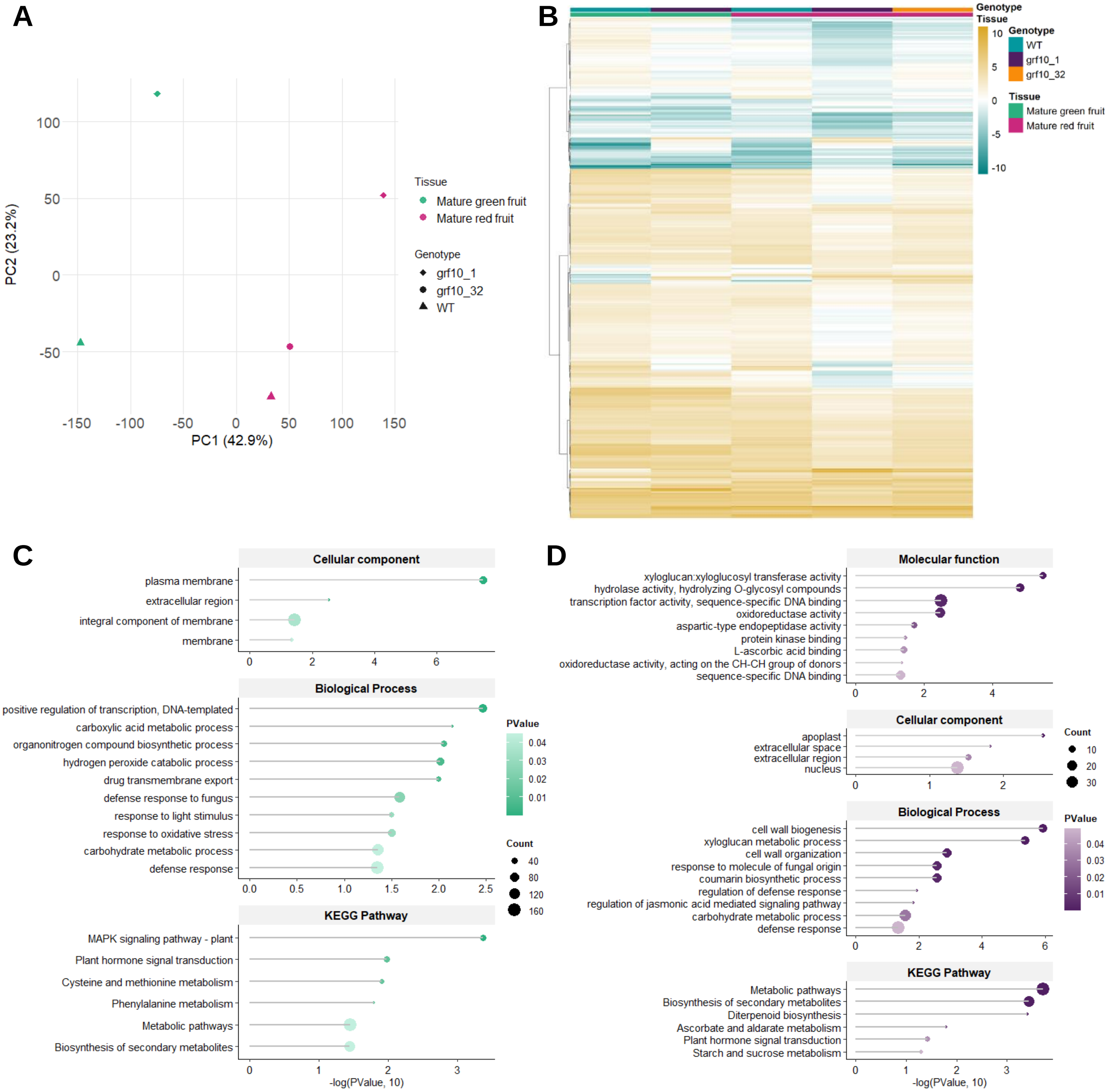

Supplementary Figure S14. Transcriptomic analysis of *grf10* shows an enrichment in several gene ontology (GO) terms and KEGG pathways. A) Principal component analysis of 25,021 transcripts of averaged triplicates of mature green and red fruits of wild type (WT; *S. lycopersicum* cv. MoneyMaker). **B)** Heat map of significantly differentially expressed genes (Benjamini Hochberg adjusted p-value < 0.05 calculated by either Student's *t*-test or Wilcoxon rank test based on the normal distribution and $|\log_2$ fold change$| > 1$. **C)** GO terms and KEGG pathways enriched in differentially expressed genes ($|\log_2 FC| > 1$) of mature green and **D)** red fruits of *grf10_1* compared to wild type.

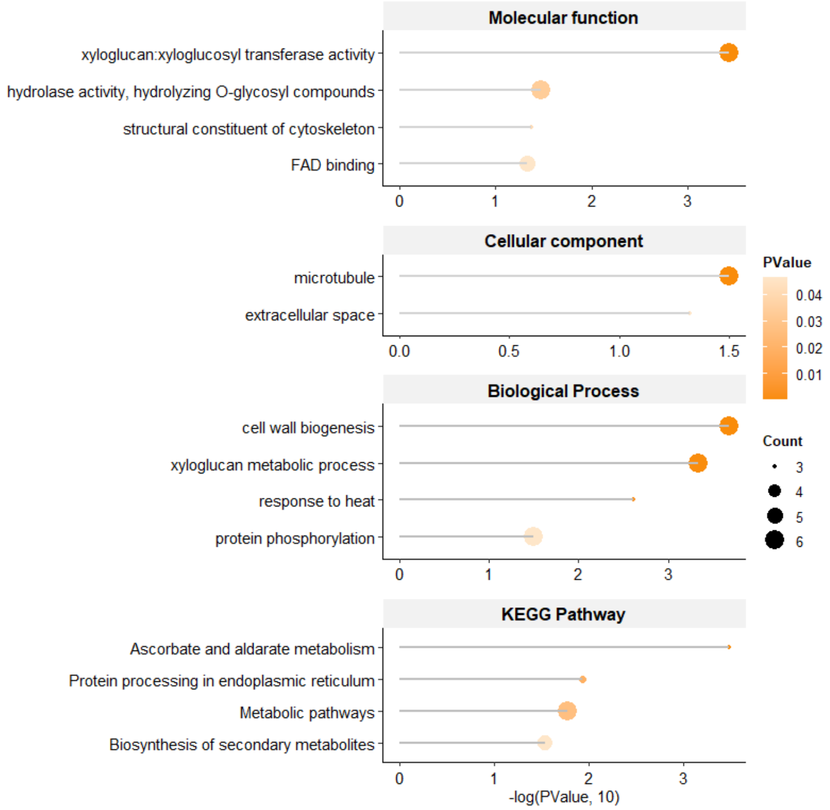

Supplementary Figure S15. Transcriptomic analysis of mature red wild type fruits versus heterozygous T_0 *grf10_32* fruits. GO terms and KEGG pathways enriched in differentially expressed genes ($|log_2FC| > 1$) of wild type *S. lycopersicum* cv. MoneyMaker mature red fruits versus mature red fruits of *grf10_32* using DAVID (http://david.abcc.ncifcrf.gov/).

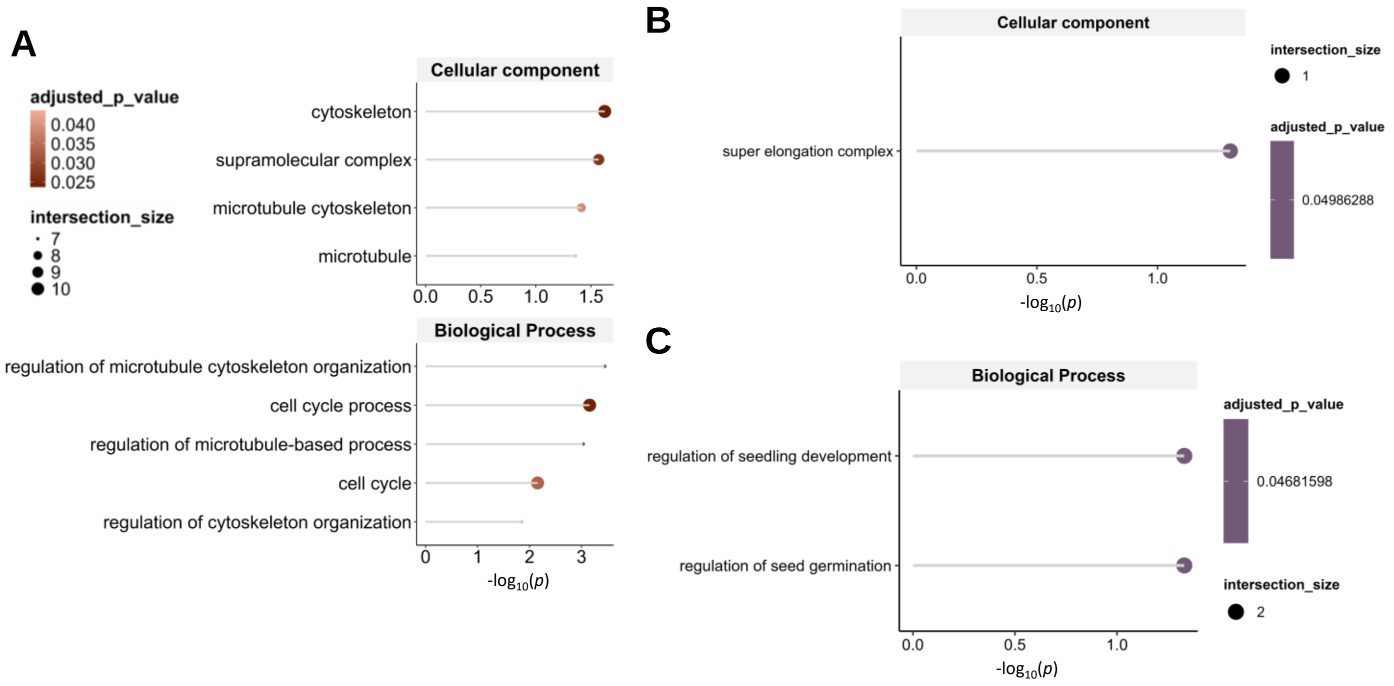

Supplementary Figure S16. Gene ontology enrichment analysis of transcriptomic data 7 and 20 days-post anthesis (dpa) from T_3. GO enrichment **A)** for all transcripts 7 dpa ($|log_2FC| > 1$), **B)** for downregulated transcripts 20 dpa ($log_2FC < -1$) and **C)** for upregulated transcripts 20 dpa ($log_2FC > 1$).
